# Immunoinformatic Design and Evaluation of a Multi-Epitope mRNA Vaccine RP14914P Targeting Latent Tuberculosis Infection

**DOI:** 10.3390/pathogens15030297

**Published:** 2026-03-09

**Authors:** Yuan Tian, Mingming Zhang, Syed Luqman Ali, Aigul Abduldayeva, Shuang Zhou, Yajing An, Yufeng Li, Ruizi Ni, Lingxia Zhang, Yanhua Liu, Weiguo Sun, Wenping Gong

**Affiliations:** 1 Senior Department of Tuberculosis, Chinese PLA General Hospital, Beijing 100091, China; tianyuan@stu.sqxy.edu.cn (Y.T.); zhangmingming@stu.sqxy.edu.cn (M.Z.); zhoushuang@stu.sqxy.edu.cn (S.Z.); anyajing@asu.edu.pl (Y.A.); liyufeng@stu.sqxy.edu.cn (Y.L.); ruizini@asu.edu.pl (R.N.); 1707025046@stu.sqxy.edu.cn (L.Z.); 1707025045@stu.sqxy.edu.cn (Y.L.); 2 Graduate School, Hebei North University, Zhangjiakou 075000, China; 3 Department of Biochemistry, Abdul Wali Khan University, Mardan 23200, Pakistan; syedluqmanali5@gmail.com; 4 Department of Research Institute of Preventive Medicine Named Academician E. Dalenov, Astana Medical University, Astana 010000, Kazakhstan; abduldayeva.a@amu.kz; 5 Department of Geriatrics, The Eighth Medical Center of PLA General Hospital, Beijing 100091, China

**Keywords:** multi-epitope vaccine, immunoinformatics, reverse vaccinology, molecular docking, immune simulation, vaccine design

## Abstract

**Background**: Latent tuberculosis infection (LTBI) is the principal reservoir for active tuberculosis, with >85% of cases attributable to reactivation. Bacillus Calmette-Guérin fails to block this transition, leaving a critical gap in prevention. **Methods**: An immunoinformatics/reverse-vaccinology pipeline was applied to seven dormancy-related antigens retrieved from Mycobrowser. T-cell epitopes were predicted with NetMHCI/IIpan-4.1 and B-cell epitopes with ABCpred; antigenicity, allergenicity, and toxicity were evaluated with VaxiJen, AllerTOP, and ToxinPred. Secondary/tertiary structures were modeled with PSIPRED and AlphaFold-3; docking to Toll-like receptors (TLR) 2/4 and 100 ns molecular dynamics simulations assessed complex stability. Immune responses were simulated with C-ImmSim, and the mRNA sequence was human-codon-optimized using ExpOptimizer. **Results**: The resulting construct, RP14914P, encodes 14 cytotoxic T lymphocyte, 9 helper T lymphocyte, and 14 B-cell epitopes within an 866-aa, 90.4 kDa polypeptide. Antigenicity score = 0.7797, immunogenicity score = 8.58629. and no toxicity or allergenicity was predicted. Physicochemical analysis: instability index = 28.65, and solubility = 0.513. Estimated population coverage is 82.35% and 99.67% for Human Leukocyte Antigen (HLA)-I and HLA-II globally. Docking energies: −1477.8 kcal/mol (TLR2) and −1480.1 kcal/mol (TLR4). Molecular dynamics trajectories confirm stable binding. Immune simulation predicts potent activation of Natural Killer cells, macrophages, and dendritic cells, Th1 polarization, high interferon-γ/interleukin-2 secretion, and durable memory. **Conclusions**: In silico analyses predict that RP14914P exhibits favorable immunogenicity, safety, and broad population coverage, suggesting its potential as a promising mRNA vaccine candidate to prevent LTBI reactivation. However, these computational predictions require thorough experimental validation to confirm the vaccine’s immunogenicity and protective efficacy.

## 1. Introduction

Tuberculosis (TB), a chronic infectious disease caused by agents of the *Mycobacterium tuberculosis* complex (MTBC), remains a major global health threat [1,2]. Its control is critically undermined by the high prevalence and reactivation risk of latent tuberculosis infection (LTBI) [3,4,5,6,7]. According to the WHO Global Tuberculosis Report 2025, an estimated 10.7 million incident cases of active tuberculosis (ATB) and 1.23 million deaths occurred worldwide in 2024, and China alone contributed ~696,000 cases and 25,000 deaths [8]. Approximately one-quarter of the world’s population—nearly two billion individuals—harbor MTBC in a latent state. These asymptomatic carriers are at a lifelong risk of reactivation when immunity wanes, and >85% of ATB cases are attributed to LTBI reactivation [9]. This “latency-to-activation” cycle constitutes the central obstacle to TB eradication, and current interventions leave a decisive technological gap.

The only licensed vaccine, Bacillus Calmette-Guérin (BCG), protects infants against disseminated TB but shows highly variable efficacy (0–80%) in adolescents and adults [3,10,11]. During the past two decades, candidate vaccines entering clinical evaluation (e.g., M72/AS01E, H56:IC31, and GamTBvac) have focused on preventing primary disease or boosting BCG-primed immunity; none is explicitly designed to interrupt LTBI reactivation. Moreover, most formulations rely on antigens expressed during active replication (e.g., Ag85B and ESAT-6) and therefore provide limited coverage of the diverse antigenic repertoire displayed across MTBC’s multi-stage life-cycle [12,13,14,15]. Closing the “LTBI-to-ATB” bottleneck thus represents a pivotal strategic unmet need.

The rapid advancement of immunoinformatics and mRNA technology offers a revolutionary opportunity to address this gap systematically [16,17,18,19,20,21,22]. Compared to traditional platforms, mRNA vaccines possess distinct advantages: (i) design flexibility allows for the integration of multiple protective epitopes from different stages into a single transcript, enabling comprehensive, multi-stage targeting of the MTBC life-cycle; (ii) endogenously expressed antigens facilitate simultaneous activation of both major histocompatibility complex (MHC)-I and MHC-II pathways, synergistically inducing CD8^+^ Cytotoxic T Lymphocyte (CTL) and CD4^+^ Helper T cell (Th1) responses, thereby overcoming the limitations of current vaccines in eliciting robust CTL activity; and (iii) the platform allows rapid adaptation to pathogen variations through “programmable” sequence iterations to optimize population coverage and immunogenicity [23,24]. Notably, BNT164a1 and BNT164b1 have entered Phase I clinical trials, marking a transition of TB mRNA vaccines from proof-of-concept to clinical translation [11]. However, an mRNA vaccine specifically targeting the LTBI stage, systematically integrating multiple epitopes, and possessing broad population coverage potential is still lacking.

**Figure 1:**
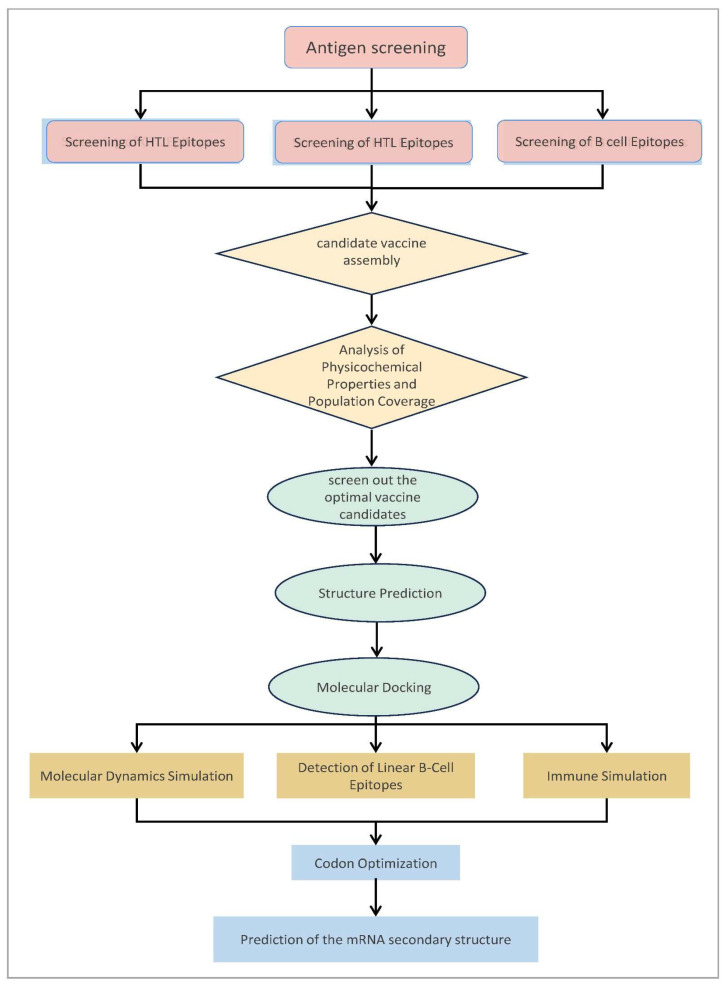
Workflow of the immunoinformatics pipeline for RP14914P. The flowchart delineates every computational step, from mining latency antigens to final mRNA construct evaluation.

Based on this research landscape and technological need, this study employed immunoinformatics and reverse vaccinology strategies to undertake the following work (Figure 1): First, dominant epitopes capable of concurrently eliciting CTL, Helper T Lymphocyte (HTL), and B-cell responses were screened from key latency-associated antigens (Rv1736c, Rv1980c, Rv2656c, Rv2659c, Rv3872, Rv3873, and Rv3879c) of *Mycobacterium tuberculosis* (MTB) H37Rv strain, with their safety validated through predictions of antigenicity, allergenicity, and toxicity. Second, Toll-like receptors (TLR) 2/4 agonists and the PADRE helper peptide were incorporated as built-in adjuvants, and a multi-epitope mRNA vaccine (designated RP14914P) was assembled using specific linkers (AAY, GPGPG, and KK). Finally, comprehensive in silico evaluations were conducted to assess the vaccine’s physicochemical properties, global population coverage, secondary and tertiary structure stability, molecular interactions with TLRs, and simulated immune response profiles. This study aims to design a broad-spectrum, safe, and efficacious multi-epitope mRNA vaccine targeting LTBI, providing a novel candidate molecule to prevent the progression from LTBI to ATB, while also offering a technical reference for the rational design of TB mRNA vaccines.

## 2. Materials and Methods

### 2.1. Antigen Selection and Sequence Retrieval

Based on an extensive literature review and our prior research experience, we selected seven antigens—Rv1736c, Rv1980c, Rv2656c, Rv2659c, Rv3872, Rv3873, and Rv3879c (Table 1) [25,26,27,28,29,30]—from a pool of 21 latency-associated antigens for subsequent analysis. The selection criteria were: (i) confirmed expression during MTB latency; (ii) potential immunogenicity; and (iii) being in preclinical or clinical research stages, indicating a foundational level of prior investigation. The FASTA format amino acid sequences for all candidate antigens were retrieved from the Mycobrowser database (https://mycobrowser.epfl.ch/, accessed on 11 February 2025).

### 2.2. Prediction and Screening of Immunodominant Epitopes

To construct a multi-epitope vaccine capable of eliciting a comprehensive immune response, we systematically predicted and screened CTL, HTL, and B-cell epitopes.

#### 2.2.1. CTL Epitope Prediction

CTL epitopes were predicted using the MHC-I binding prediction tool within the IEDB database (https://nextgen-tools.iedb.org/pipeline?tool=tc1, accessed on 25 February 2025) [31]. The NetMHCpan-4.1 EL (recommended epitope predictor-2023.09) and NetMHCpan-4.1 BA (recommended binding predictor-2023.09) algorithms were employed to predict 9-10mer peptides against the complete human leukocyte antigen (HLA) reference allele set. Initial screening criteria were: EL rank < 0.5% and BA IC50 < 50 nM. Subsequently, a secondary filter was applied, requiring: antigenicity (VaxiJen v2.0) > 0.5, immunogenicity (IEDB Immunogenicity tool) > 0, non-toxicity (ToxinPred2.0), and non-allergenicity (AllerTOP v.2.1). Duplicate and similar sequences were removed.

#### 2.2.2. HTL Epitope Prediction

HTL epitopes were predicted using the IEDB MHC-II binding prediction tool (https://nextgen-tools.iedb.org/pipeline?tool=tc2, accessed on accessed on 25 February 2025) [32] with the NetMHCIIpan-4.1 EL algorithm, predicting 12-18mer peptides against the full HLA reference allele list. Initial screening criteria were: EL rank < 0.5% and antigenicity > 0.5. The secondary filtering criteria were identical to those for CTL epitopes. Subsequently, the IFNepitope (https://webs.iiitd.edu.in/raghava/ifnepitope/design.php, accessed on 25 February 2025) [33], IL4pred (https://webs.iiitd.edu.in/raghava/il4pred/index.php, accessed on 25 February 202525) [34], and IL10pred (https://webs.iiitd.edu.in/raghava/il10pred/predict3.php, accessed on 25 February 2025) [35] online servers were used to evaluate the epitopes’ ability to induce specific cytokines. Epitopes predicted to induce IFN-γ-positive responses, while not inducing interleukin-4 (IL-4) and IL-10 secretion, were prioritized to bias the immune response towards a Th1 phenotype.

#### 2.2.3. B-Cell Epitope Prediction

Linear B-cell epitopes were predicted using the ABCpred server (https://webs.iiitd.edu.in/raghava/abcpred, accessed on 27 February 2025) [36], which employs a recurrent neural network. The threshold was set to 0.51, and the two highest-scoring epitopes from each antigen were selected.

#### 2.2.4. Epitope Homology Prediction

To gauge the vaccine’s potential for cross-strain protection, we mapped the evolutionary conservation of every CTL, HTL, and B-cell epitope it contains. Using BLASTP (https://blast.ncbi.nlm.nih.gov/Blast.cgi, accessed on 12 January 2026) on the NCBI portal, we restricted the search space to the genus *Mycobacterium* (taxonomy ID: 1763) and queried each peptide in turn, quantifying its percent identity to the major lineages of the MTBC, as well as to selected non-tuberculous mycobacteria (NTM).

#### 2.2.5. Epitope Safety Validation

All final selected epitopes underwent safety validation using VaxiJen v2.0 (https://www.ddg-pharmfac.net/vaxijen/VaxiJen/VaxiJen.html, accessed on 5 March 2025) [37] for antigenicity, AllerTOP v.2.1 (https://www.ddg-pharmfac.net/allertop_test/, accessed on 7 March 2025) [38] for allergenicity, and ToxinPred2.0 (https://webs.iiitd.edu.in/raghava/toxinpred2/algo.html, accessed on 7 March 2025) [39] for toxicity, ensuring compliance with safety standards for vaccine components.

### 2.3. mRNA Vaccine Construct Assembly

The selected epitopes were tandemly assembled using appropriate amino acid linkers. To optimize antigen processing and presentation and minimize the generation of junctional epitopes, the AAY, GPGPG, and KK linkers were used to connect CTL, HTL, and B-cell epitopes, respectively. To enhance vaccine immunogenicity, a TLR2 agonist, a TLR4 agonist, and the universal helper peptide (PADRE) were incorporated at the N-terminus via an EAAAK rigid linker. Finally, a 6×His tag was added to the C-terminus to facilitate subsequent protein purification and identification. For mRNA vaccine production, the final mRNA vaccine construct was designed from 5′ to 3′ as follows: 5′ m7G Cap—5′ UTR—Kozak sequence—TPA (signal peptide)—EAAAK (linker)—Psmα4 (adjuvant)—GPGPG (linker)—PADRE—GPGPG/AAY (linker)—CTL epitopes—GPGPG—HTL epitopes—KK (linker)—B-cell epitopes—EAAAK—Rpfe (adjuvant)—6×His—MITD sequence—3′ UTR—Poly(A) tail [23,24,40,41].

### 2.4. Analysis of Physicochemical Properties, Antigenicity, and Safety of the Candidate Vaccine

The theoretical molecular weight, isoelectric point (pI), instability index, aliphatic index, and grand average of hydropathicity (GRAVY) of the vaccine construct were predicted using the ExPASy ProtParam tool (https://web.expasy.org/protparam, accessed on 12 March 2025) [42]. Its solubility in *E. coli* was predicted using the Protein-sol server (https://protein-sol.manchester.ac.uk, accessed on 10 March 2025) [43], with a value >0.45 considered indicative of good solubility. Antigenicity and immunogenicity were predicted using VaxiJen v2.0 and the IEDB Immunogenicity tool, respectively. Allergenicity and toxicity were assessed using AllerTOP v.2.1 and ToxinPred2.0.

### 2.5. Selection of the Optimal Vaccine Construct

To identify a superior candidate, the screened CTL, HTL, and linear B-cell epitopes were randomly combined. The resulting candidate vaccines were evaluated based on biological characteristics, physicochemical properties, and solubility, leading to the identification of the optimal set of CTL, HTL, and B-cell epitopes. These were then randomly combined with different TLR agonists and the helper peptide. The optimal combination of agonists and helper peptide was determined, and this final construct was designated RP14914P.

### 2.6. Secondary and Tertiary Structure Prediction and Validation

The secondary structure of the vaccine was predicted using the PSIPRED (http://bioinf.cs.ucl.ac.uk/psipred, accessed on 2 April 2025) and SOPMA (https://npsa.lyon.inserm.fr/cgi-bin/npsa_automat.pl?page=/NPSA/npsa_sopma.html, accessed on 2 April 2025) servers [44,45,46]. The tertiary structure was predicted using AlphaFold-3 (https://golgi.sandbox.google.com, accessed on 5 April 2025), and the best model was selected from the five generated. The preferred tertiary structure model was subsequently refined using GalaxyRefine (https://galaxy.seoklab.org, accessed on 10 April 2025). The refined model was validated using ProSA-web (https://prosa.services.came.sbg.ac.at/prosa.php, accessed on 11 April 2025) (Z-score), ERRAT (https://saves.mbi.ucla.edu, accessed on 10 April 2025) (structure quality), and UCLA-DOE LAB-SAVES v6.1 (https://saves.mbi.ucla.edu, accessed on 17 April 2025) (Ramachandran plot) [47,48,49,50,51].

### 2.7. Immunoinformatics and Interaction Analysis

#### 2.7.1. Population Coverage Analysis

The projected population coverage of the selected MHC class I and II epitopes across different geographical regions was calculated using the IEDB population coverage calculation tool (http://tools.iedb.org/population, accessed on 25 April 2025) [52].

#### 2.7.2. Discontinuous B-Cell Epitope Prediction

Conformational B-cell epitopes were predicted using the ElliPro server (http://tools.iedb.org/ellipro, accessed on 14 April 2025) with a threshold score set to >0.735 [53].

#### 2.7.3. Molecular Docking

The crystal structures of TLR2 (PDB ID: 2Z7X) and TLR4 (PDB ID: 3FXI) were retrieved from the PDB database. Rigid body molecular docking between the vaccine (RP14914P) and TLR2/TLR4 was performed using the ClusPro 2.0 online server (https://cluspro.bu.edu, accessed on 22 April 2025) [54,55]. The cluster with the lowest binding energy was selected from the generated models for visualization analysis using PyMOL (Version 3.1.0) [56] and interaction analysis using LigPlot+ (Version 2.2) [57].

#### 2.7.4. Molecular Dynamics (MD) Simulation

Here, 100 ns MD simulations were performed for the RP14914P-TLR2 and RP14914P-TLR4 complexes using the Desmond module of the Schrödinger Suite [58]. The systems were equilibrated under the OPLS4 force field in an NPT ensemble at 300 K and 1 atm. Trajectory analysis included calculating the root mean square deviation (RMSD), root mean square fluctuation (RMSF), and radius of gyration (Rg). Principal component analysis (PCA) and free energy landscape (FEL) analysis were performed based on Cα atom trajectories using GROMACS. The dynamic cross-correlation matrix (DCCM) was calculated using the Bio3D R package (Version 3.1.0).

#### 2.7.5. Immune Simulation

The immune response elicited by the vaccine was simulated using the C-ImmSim server (https://150.146.2.1/C-IMMSIM/index.php, accessed on 25 June 2025) [59]. The simulation volume was set to 50, with three injections (time steps: 1, 90, and 180), and a total of 1050 steps (each step representing 8 h); other parameters were kept at default.

### 2.8. Codon Optimization and mRNA Structure Prediction

To optimize RP14914P expression in *Homo sapiens*, codon optimization was performed using the NovoPro ExpOptimizer tool (https://www.novoprolabs.com/tools/codon-optimization, accessed on 28 June 2025) [60], aiming for a Codon Adaptation Index (CAI) close to 1.0 and a GC content within the ideal range of 30–70%. The secondary structure and minimum free energy (MFE) of the optimized mRNA sequence were predicted using the RNAfold tool from the ViennaRNA Package 2.0 (http://rna.tbi.univie.ac.at, accessed on 6 August 2025) [61].

## 3. Results

### 3.1. Rational Design and Assembly of the Multi-Epitope mRNA Vaccine RP14914P

**Figure 2:**
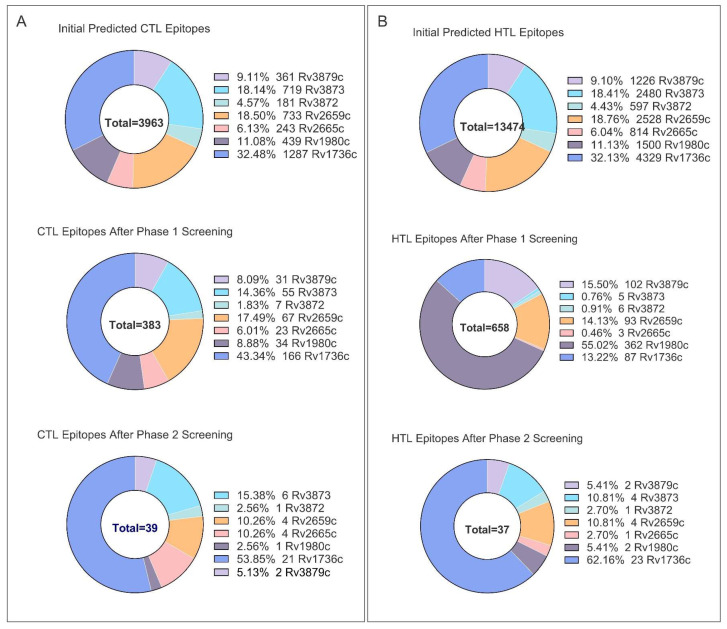
NetMHCpan-4.1 prediction and short-listing of MHC-I (**A**) and MHC-II (**B**) epitopes for the seven selected latency-associated antigens.

Based on the construction strategy illustrated in Figure 1, to obtain the optimal antigen epitopes, a three-step screening strategy was implemented as follows:

For CTL epitopes, an initial screening was performed using the EL rank and IC_50_ values generated by the NetMHCpan-4.1 tool, which yielded 383 epitopes from an initial pool of 3963 candidates (Figure 2A). Subsequent fine screening was conducted based on the criteria of antigenicity, immunogenicity, non-toxicity, and non-allergenicity, resulting in 39 epitopes (Figure 2A). After removing redundant epitopes, a final set of 31 CTL epitopes was obtained (Table S1).

For HTL epitopes, 658 epitopes were first selected on the basis of satisfying binding affinity and antigenicity requirements (Figure 2B). Further screening was then carried out to identify epitopes capable of inducing IFN-γ secretion without triggering the production of IL-4 and IL-10. This process yielded 37 HTL epitopes with a bias toward Th1-type immune responses (Figure 2B), which were further reduced to 13 unique epitopes after eliminating redundancy (Table S2).

In addition, 60 potential epitopes were predicted using the ABCpred server, from which the top two highest-scoring linear B-cell epitopes were selected per antigen (Table S3).

As outlined above, 31 dominant CTL, 13 HTL, and 14 B-cell epitopes survived the multi-step filtering pipeline. Because epitope order can shape immunogenicity [62,63], we next generated 20 topological scaffolds in which these lead epitopes were shuffled at random (Table S4). Comparative profiling of their biological behavior, physicochemical fingerprints, and predicted solubility identified scaffold No.13—carrying 14 CTL, 9 HTL, and 14 B-cell epitopes—as the top performer (Table 2), and it was advanced as the vaccine backbone.

**Figure 3:**
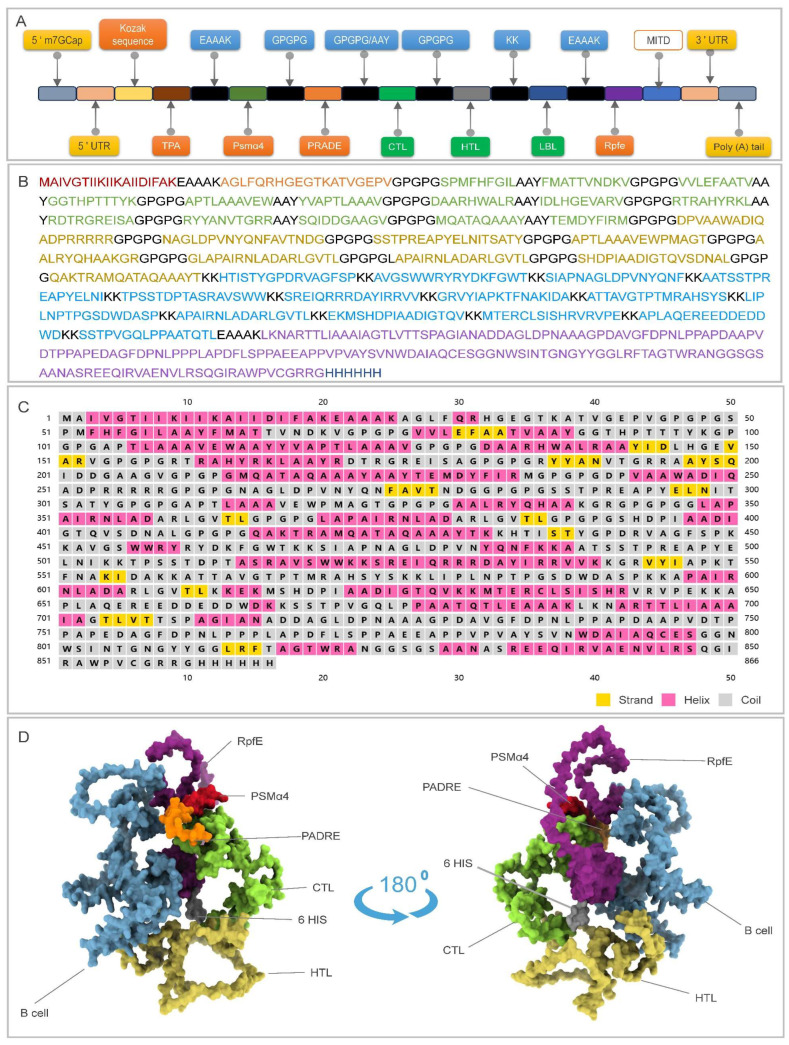
Architectural overview of RP14914P. (**A**) Schematic of the mRNA cassette: 5′-m7GCap-5′UTR-Kozak-TPA-EAAAK-Psmα4-GPGPG-PADRE-GPGPG/AAY-CTLs-GPGPG-HTLs-KK-B-cells-EAAAK-Rpfe-6×His-MITD-3′UTR-poly(A). (**B**) Color-coded primary sequence highlighting each immunological module. (**C**) Secondary-structure prediction (PSIPRED): α-helices (pink), β-strands (yellow), and coils (gray). (**D**) AlphaFold-3 tertiary model visualized in ChimeraX 1.8.

Built upon scaffold No.13, we installed four TLR2 and five TLR4 agonists and, by permuting their positions, obtained 26 candidate molecules (Table S5). Ranking these constructs on antigenicity, immunogenicity, toxicity, allergenicity, molecular weight, theoretical pI, instability index, aliphatic index, GRAVY, and solubility singled out the combination of the TLR2 agonist Psmα4 and the TLR4 agonist Rpfe. This pair was grafted onto scaffold No.13 to yield the final mRNA vaccine, designated RP14914P (Figure 3A,B).

### 3.2. Prediction and Optimization of Secondary and Tertiary Structures

Secondary-structure prediction indicates 27.25% α-helix, 12.70% extended strand, and 60.05% random coil, offering an optimal balance between rigidity and surface plasticity for immune recognition (Figure 3C). AlphaFold-3 generated its tertiary model (Figure 3D).

**Figure 4:**
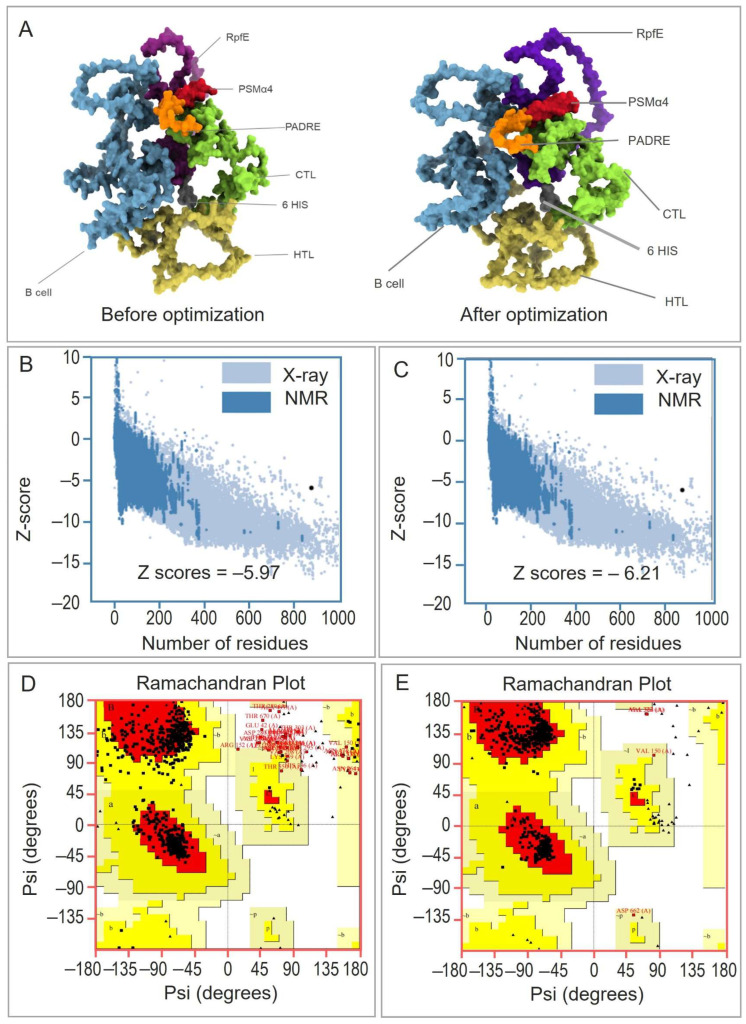
Structural optimization and validation of the RP14914P vaccine model. (**A**) Cartoon representations of the tertiary structure before (**left**) and after (**right**) refinement using the GalaxyRefine server. The model is colored by functional module in sequential order from N- to C-terminus: TLR2 agonist PSMα4 (red), universal helper peptide PADRE (orange), CTL epitopes (green), HTL epitopes (yellow), B-cell epitopes (blue), and TLR4 agonist RpfE (purple). (**B**,**C**) ProSA-web Z-score plots for the model before (**B**, Z-score = −5.97) and after (**C**, Z-score = −6.21) refinement, indicating improved overall model quality relative to known protein structures (X-ray and NMR). (**D**,**E**) Ramachandran plots for the model before (**D**) and after (**E**) optimization. Residues in the most favored, additionally allowed, generously allowed, and disallowed regions are colored dark red, yellow, light yellow, and white, respectively.

To ensure the reliability of the 3D structure prediction, we used the Galaxy Refine server to perform structural optimization (Figure 4A). The generated models were evaluated based on GDT-HA, RMSD, MolProbity, Clash score, and Poor rotamers. Model 5 (Table S6) was selected as the final 3D model of the vaccine. The z-scores before and after optimization were −5.97 (Figure 4B) and −6.21 (Figure 4C), respectively, indicating that the quality of the model was improved after optimization.

In addition, Ramachandran plot analysis revealed that prior to optimization, 83.1% of the amino acid residues fell within the favored regions, 11.4% within the additionally allowed regions, 3.6% within the generously allowed regions, and 1.9% within the disallowed regions (Figure 4D). After optimization, the proportion of residues in the favored regions increased to 97.5%, with 1.9% in the additionally allowed regions, 0.3% in the generously allowed regions, and 0.3% in the disallowed regions (Figure 4E). These results further confirm the effectiveness of the model optimization process.

### 3.3. Favorable Physicochemical Profile, Safety, and Global Population Coverage of RP14914P

In silico profiling revealed robust antigenicity (VaxiJen 0.7797) and immunogenicity (IEDB score 8.58629), with no predicted allergenicity or toxicity. The protein exhibits a theoretical pI of 9.61, an instability index of 28.65 (<40 threshold), an aliphatic index of 67.27, and a GRAVY of −0.424, indicating a hydrophilic and stable molecule. Protein-sol predicted an *E. coli* solubility score of 0.513 (>0.45 cut-off), and estimated half-lives are 30 h (mammalian reticulocytes), 20 h (yeast), and 10 h (*E. coli*).

**Figure 5:**
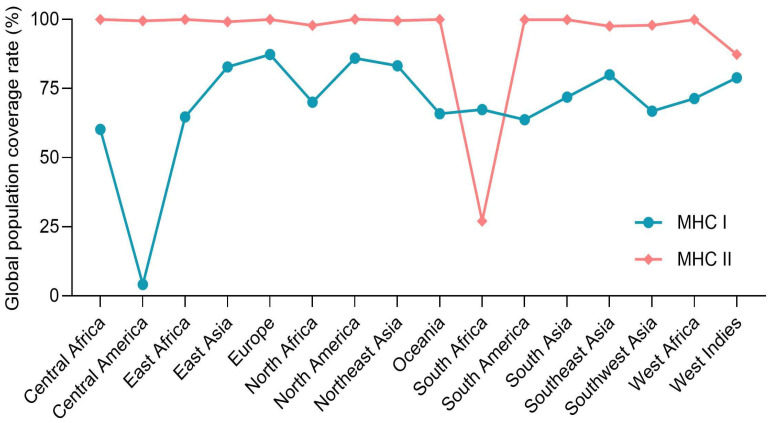
Population coverage of selected MHC epitopes. Global and regional coverage distributions for HLA-I and HLA-II alleles are displayed.

Allele frequency-weighted coverage analysis demonstrated that included MHC-I epitopes span 82.35% of the global population (peak 85.97% in North America), whereas MHC-II epitopes cover 99.67% (peak 100% in North America) (Figure 5), underscoring the construct’s broad ethnic applicability.

### 3.4. Conservation Analysis of Vaccine Epitopes Across Mycobacterium Strains

**Figure 6:**
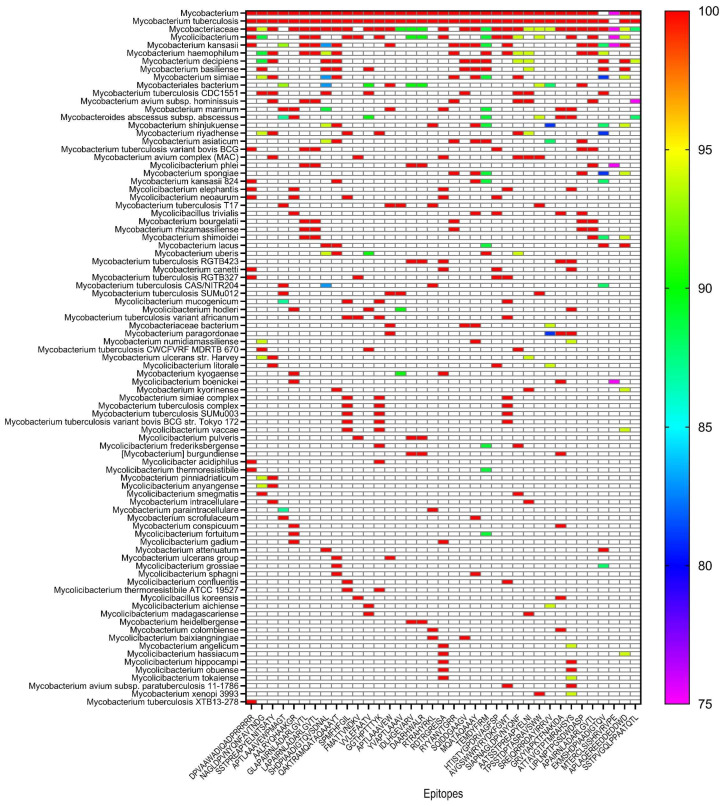
Conservation landscape of the RP14914P vaccine epitopes across MTBC and NTM strains. Heatmap showing the percentage of sequence identity for each of the 37 vaccine epitopes (*x*-axis) against 87 MTBC and NTM strains (*y*-axis). Epitopes are grouped by type (HTL, CTL, and B-cell). Identity values are color-coded from red (high identity) to purple (low identity), based on pairwise BLASTP analysis. White cells indicate that no significant homology was detected for the epitope in the corresponding strain.

To evaluate the potential of the RP14914P vaccine to confer broad-spectrum immunity, we systematically analyzed the sequence conservation of its 37 core epitopes across 87 Mycobacterium strains, including major branches of the MTBC and clinically relevant NTM. The results are visualized as a heatmap (Figure 6).

BLAST revealed that the core immunogenic epitopes of the vaccine exhibited excellent cross-strain stability: 19 epitopes (approximately 51% of the total) showed 100% sequence identity in 87 tested strains, corresponding to large continuous deep-red regions in the heatmap. This indicates that these epitopes are fully conserved across a broad range of pathogen lineages, including major MTBC branches and multiple NTM species (e.g., *Mycobacterium avium* complex), providing a structural basis for the vaccine’s broad-spectrum protection.

The remaining 16 epitopes maintained high sequence identity (81–100%): 14 of these epitopes retained ≥80% identity across all strains. Only two B-cell epitopes (MTERCLSISHRVRVPE and SSTPVGQLPPAATQTL) exhibited <90% sequence identity, corresponding to blue-to-purple regions in the heatmap, with the lowest value being 75%. This suggests mild variation in these two epitopes in some strains, while their overall homology remains high.

Collectively, the 37 epitopes of the RP14914P vaccine demonstrated high conservation across 87 MTBC and NTM strains, with only minor variations in a small subset of epitopes. These findings strongly support the potential of the vaccine to induce broad-spectrum immune protection.

### 3.5. Prediction of Discontinuous B-Cell Epitopes

ElliPro discontinuous epitope mapping (threshold 0.735) identified 20 conformational B-cell epitopes (scores 0.735–0.962) distributed across the molecular surface (Table S7), implying potent capacity to elicit high-affinity antibody responses.

### 3.6. High-Affinity Docking to TLR2/4 and Robust MD Trajectories

**Figure 7:**
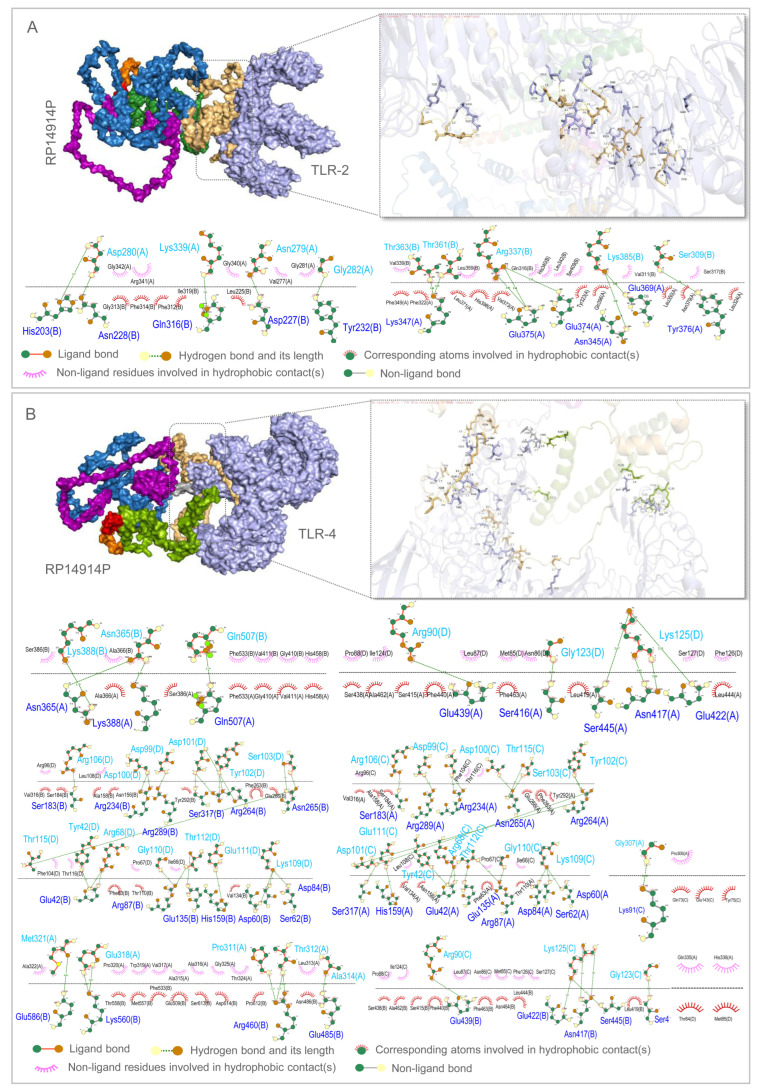
Molecular docking of RP14914P with TLR2 (**A**) and TLR4 (**B**). Left panels: ClusPro lowest-energy poses; right panels: magnified interaction zones; bottom panel: LigPlot 2D maps detailing hydrogen bonds and hydrophobic contacts.

**Figure 8:**
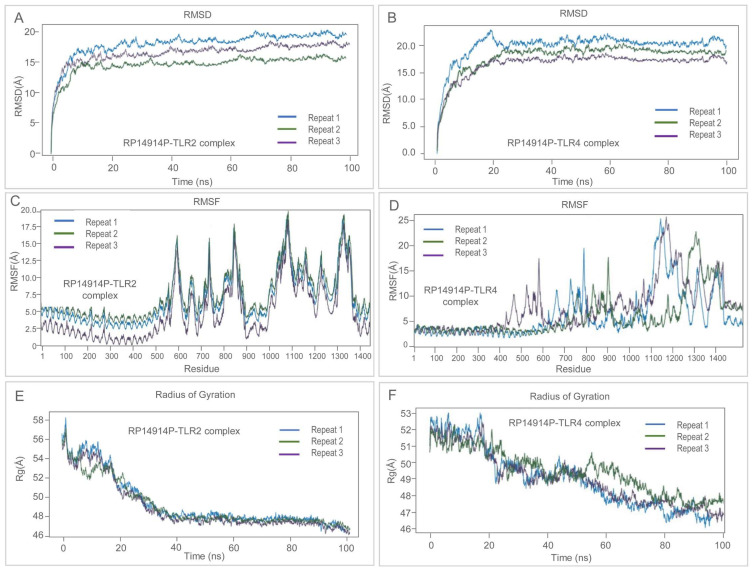
Molecular dynamics simulation analysis of the RP14914P vaccine construct in complex with TLR2 and TLR4 over 100 ns, performed in three independent repeats. (**A**) Backbone RMSD of the RP14914P–TLR2 complex and (**B**) RP14914P–TLR4 complex, showing initial equilibration followed by stable trajectories across all repeats. (**C**) RMSF profiles of the RP14914P–TLR2 complex and (**D**) RP14914P–TLR4 complex, illustrating residue-wise flexibility, with higher fluctuations primarily confined to loop and terminal regions. (**E**) Radius of gyration (Rg) of the RP14914P–TLR2 complex and (**F**) RP14914P–TLR4 complex, indicating progressive compaction and maintenance of overall structural stability throughout the simulation. Blue, green, and purple lines represent Repeat 1, Repeat 2, and Repeat 3.

**Figure 9:**
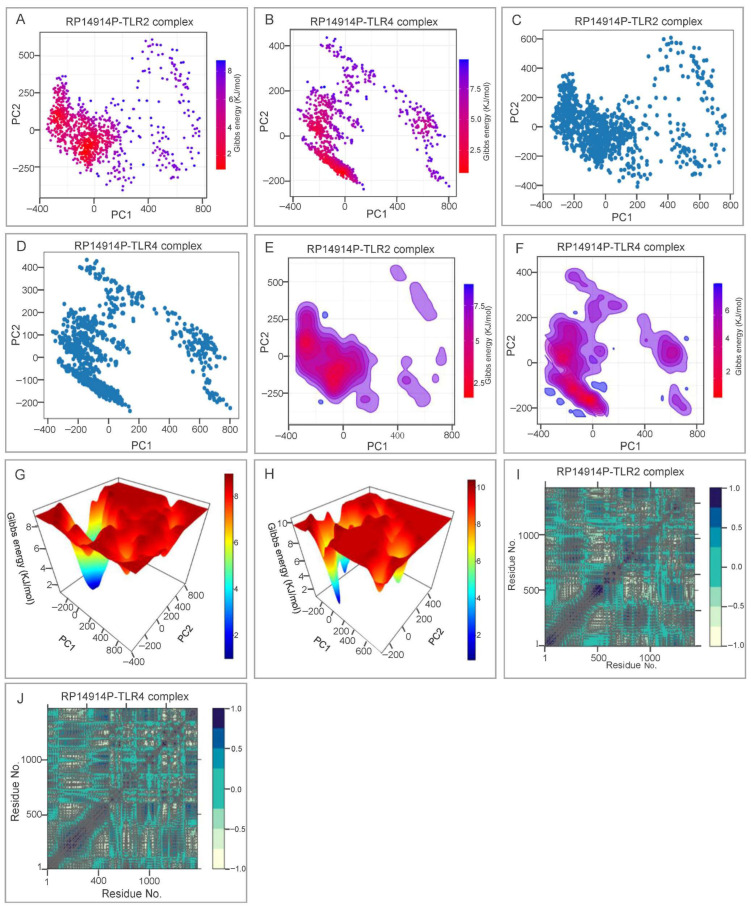
Collective motion and energetics. (**A**–**D**) PCA projections with/without Gibbs free-energy color mapping. (**E**–**H**) 2-D and 3-D free-energy landscapes revealing a single deep basin for TLR2 and multiple shallow minima for TLR4. (**I**,**J**) DCCM matrices indicating correlated (blue) and anti-correlated (brown) residue motions.

ClusPro docking returned exceptionally low binding energies of −1477.8 kcal mol^−1^ (TLR2) and −1480.1 kcal mol^−1^ (TLR4), driven by 23 and 38 hydrogen bonds, respectively (Figure 7). During 100 ns MD, both complexes equilibrated within 10 ns and displayed stable RMSD plateaus (18–23 Å). RMSF profiles revealed localized flexible loops, whereas decreasing Rg indicated progressive compaction (Figure 8). PCA and free-energy landscape analyses showed that the TLR2 complex occupies a single deep energy basin, whereas the TLR4 ensemble samples multiple shallow minima, implying greater conformational plasticity that may facilitate adaptive immune signaling (Figure 9).

### 3.7. In Silico Immune Simulation Predicts Potent Multi-Layer Immunity

**Figure 10:**
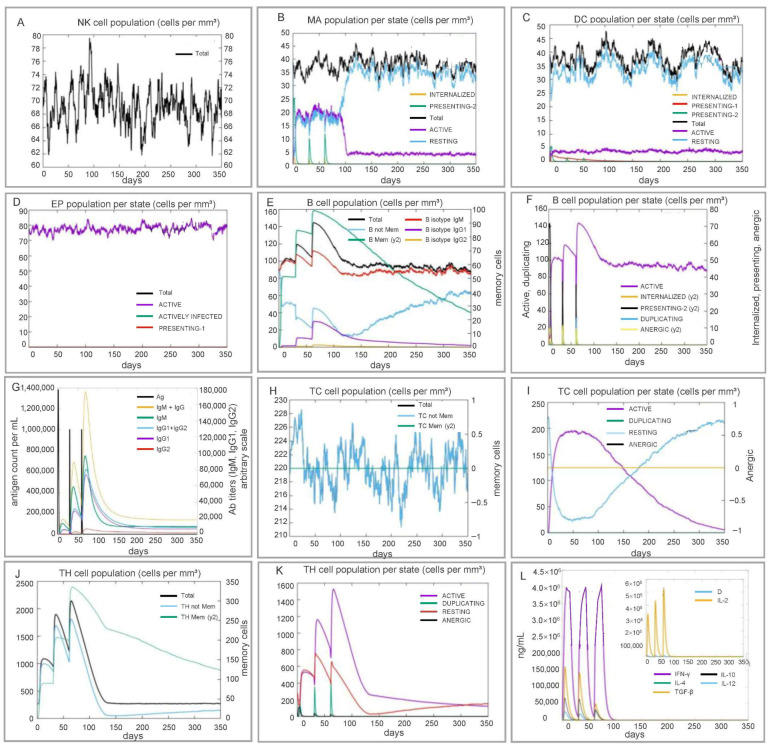
C-ImmSim immune simulation. Temporal dynamics following virtual vaccination: (**A**) NK cells, (**B**) macrophage subsets, (**C**) dendritic cells, (**D**) epithelial cells, (**E**,**F**) B-cell populations, (**G**) antibody titers, (**H**,**I**) CD4^+^ T-cell subsets, (**J**,**K**) CD8^+^ T-cell subsets, and (**L**) key cytokine profiles.

C-ImmSim projection revealed rapid and sustained activation of innate and adaptive compartments. Natural killer (NK) cells peaked at day 90 and stabilized at 62–75 cells/µL. Three waves of MHC-2 macrophages coincided with the reduction in resting macrophages, whereas activated dendritic cells steadily accumulated, reflecting efficient antigen presentation (Figure 10).

Adaptive markers showed three synchronized helper T (TH)/cytotoxic T (TC) cells activation peaks (days 10, 40, 70) and a sharp rise in memory B cells within 5–10 days, forecasting durable humoral memory. Cytokine signatures were dominated by IFN-γ and IL-2 (Th1 axis) alongside regulatory IL-10 and transforming growth factor-beta (TGF-β), predicting robust anti-mycobacterial effector function balanced by protective homeostasis.

### 3.8. Codon Optimization and mRNA Secondary-Structure Stability

**Figure 11:**
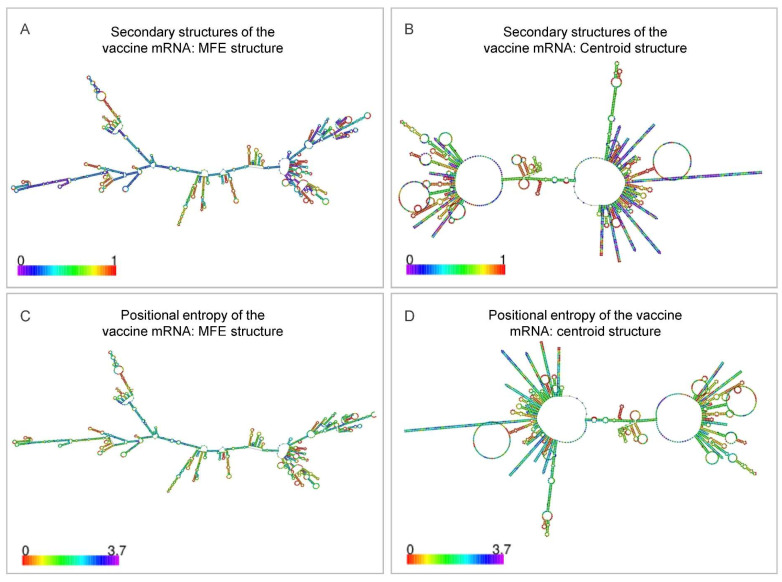
RNAfold-predicted secondary structure of the codon-optimized RP14914P mRNA. (**A**) MFE structure, (**B**) centroid structure, and corresponding positional entropy maps (**C**,**D**) illustrating thermodynamic stability and conformational flexibility.

Human codon optimization (ExpOptimizer) yielded a 2598-nt sequence with CAI = 0.81 and GC = 61.09%—parameters optimal for both structural stability and efficient translation. RNAfold predicted a MFE of −1213.9 kcal mol^−1^ for the dominant secondary structure and −648.15 kcal mol^−1^ for the centroid structure (Figure 11), confirming a thermodynamically stable yet flexible conformation compatible with high-level expression and prolonged half-life in mammalian cells.

## 4. Discussion

LTBI represents a major challenge in TB control, with approximately 85% of active TB cases stemming from the reactivation of LTBI, a process that the existing BCG vaccine fails to effectively prevent [5,64,65,66,67,68,69,70,71]. Addressing this critical issue, this study employed immunoinformatics and reverse vaccinology strategies to design and evaluate the multi-epitope mRNA vaccine RP14914P. This candidate aims to block the progression from LTBI to active TB by targeting MTB latency antigens, integrating multiple types of immune epitopes, and incorporating a dual TLR adjuvant system. The following discussion, based strictly on the data from this study and the published literature, elaborates on the design innovativeness, core characteristics, molecular mechanisms, and study limitations.

The innovative design of RP14914P is first reflected in its antigen and epitope screening strategy. Early TB mRNA vaccines often utilized a single antigen, for instance, ESAT6 [72] and MPT83 [73]. While it induced short-term protection, it did not cover latency antigens and was thus ineffective against LTBI. The Hsp65-based mRNA vaccine in 2010 shared similar limitations [74]. Even the BNT164a1/b1 vaccines, which have entered Phase I clinical trials and employ a multi-antigen design, do not explicitly incorporate LTBI-specific antigens [75,76].

In contrast, the seven latency-associated antigens selected in this study all possess the characteristics of “high expression during latency and potential for clinical translation,” aligning with the design concept of our group’s previous PP19128R vaccine [77], but with a more focused antigen selection, avoiding unnecessary antigen interference. Building on this, RP14914P integrates 14 CTL, 9 HTL, and 14 B-cell epitopes, forming a complete response chain covering both cellular and humoral immunity. Compared to the vaccine designed by Al Tbeishat et al. based on epigenetic-related proteins (containing 17 CTL, 5 HTL, and 8 B-cell epitopes) [78], our vaccine includes a greater number of B-cell epitopes, which is conducive to inducing stronger humoral immunity. Compared to our group’s ZL12138L vaccine [79], RP14914P has a further increased number of CTL and B-cell epitopes, suggesting superior potential in cytotoxic responses and antibody production.

In terms of vaccine construction strategy, RP14914P adopts a modular design, using AAY, GPGPG, and KK linkers to tandem CTL, HTL, and B-cell epitopes, respectively. This approach effectively maintains the independence of epitopes and avoids immune interference, which is consistent with multiple recent studies [40,78]. Furthermore, this study integrated TLR2/4 dual agonists and the PADRE helper peptide at the N-terminus via an EAAAK linker, reducing steric clashes while enhancing the synergy of innate immune activation. Compared to vaccines using only a single TLR adjuvant, such as the Al Tbeishat vaccine using only the TLR4 agonist RpfE [78,80] or the Shahrear et al. vaccine relying solely on the TLR2 agonist C5 peptide [23], the dual TLR agonist design of RP14914P can more comprehensively mimic natural immune recognition patterns and activate broader immune signaling pathways. Molecular docking results showed that the binding energies of RP14914P to TLR2 and TLR4 were −1477.8 kcal/mol and −1480.1 kcal/mol, respectively, significantly lower than those of our group’s previous PP19128R vaccine (−1324.77 kcal/mol and −1278 kcal/mol) and ZL12138L vaccine (−1173.4 kcal/mol and −1360.5 kcal/mol) [79], suggesting stronger binding stability and laying a structural foundation for efficient innate immune activation.

RP14914P is predicted to demonstrate promising translational potential in terms of safety, physicochemical properties, and population coverage. Its antigenicity score is 0.7797, and its immunogenicity score is 8.58629. Although slightly lower than the Al Tbeishat vaccine (antigenicity 0.814) and the PP19128R vaccine (immunogenicity 9.29811), it still meets the criteria for strong immunogenicity and has been systematically verified as non-toxic and non-allergenic. Compared to the RUTI vaccine, which has entered Phase II clinical trials and reported local adverse reactions [81], RP14914P holds an advantage in safety. Regarding physicochemical properties, its instability index is 28.65, lower than that of the Sharma vaccine (28.23) and the Al Tbeishat vaccine (33.51), indicating superior in vitro stability. Its solubility of 0.513, although lower than that of PP19128R (0.900675), still meets the basic requirements for formulation development. Population coverage analysis revealed that RP14914P achieves global HLA class I and II coverage of 82.35% and 99.67%, respectively, superior to the Al Tbeishat vaccine (99.38%) and the PP19128R vaccine (82.24%/93.71%), demonstrating broad potential applicability.

From a structural biology perspective, the secondary structure of RP14914P consists of 27.25% alpha-helices and 60.05% random coils. This proportion ensures structural stability while allowing sufficient epitope exposure [82,83,84]. Compared to the vaccine designed by Shi et al. for central nervous system TB [40], RP14914P has a higher number of amino acids (866 vs. 383) and greater structural complexity, enabling the accommodation of more epitopes. Compared to the Sharma vaccine (alpha-helix content 31%), RP14914P has a higher proportion of random coils, which is more favorable for B-cell receptor recognition. After optimization, the Ramachandran plot of the tertiary structure showed 97.5% of residues in the most favored regions, outperforming the Sharma vaccine (88.22%) and the HP13138PB vaccine (88.22%) [85], indicating high reliability of its structural model. Molecular dynamics simulations further revealed that the RP14914P-TLR2 complex exhibits concentrated conformations and high stability, whereas the RP14914P-TLR4 complex demonstrates higher conformational flexibility. This characteristic of “coexistence of stability and dynamics” may enable it to effectively activate TLR signaling pathways in different immune microenvironments, achieving sustained and adaptive regulation of the innate immune response [86,87,88].

Immune simulation results suggested that RP14914P may elicit a multi-layered immune response: NK cells peaked at 90 days post-immunization and remained stable, and activated macrophages and dendritic cells increased continuously, demonstrating its good capacity for innate immune activation. In terms of adaptive immunity, the vaccine exhibited robust T and B lymphocyte activation capacity. The total number of helper T (TH) cells, memory TH cells, and activated TH cells increased continuously, with three response peaks observed following the three immunizations. This indicates that HTL epitopes are presented to CD4^+^ T cells via MHC class II molecules, driving the differentiation of naive T cells into TH cells and memory TH cells, and further inducing a TH1-type immune response, as evidenced by significant elevations in IFN-γ and IL-2 levels [89,90,91].

Meanwhile, the number of activated cytotoxic T cells increased continuously, reaching a peak on day 50, while the number of naive TC cells decreased correspondingly. This suggests that naive T cells are effectively differentiating into effector cells, likely triggered by the binding of CTL epitopes to CD8^+^ T cells [92,93,94].

The B lymphocyte response was also prominent: memory B cells increased sharply within 5–10 days post-immunization, and activated B cells showed the same trend, whereas naive B cells decreased accordingly. These results confirm that the vaccine can rapidly induce B cell activation, antibody production, and memory formation, laying a foundation for long-term immune protection [95,96].

These simulation results, compared to the repRNA-ID91-NLC vaccine reported by Larsen et al. [97], suggest more sustained immune activation features. Compared to the mRNA prime-protein boost strategy proposed by Rais et al. [98], the Th1-type cytokine profile simulated for RP14914P better aligns with the requirements for anti-tuberculosis immunity. Although these are only computational simulation results, they are consistent with the in vitro experimental trends of our group’s previous PP13138R [99] and PP19128R [77] vaccines, providing strong support for its immunogenic potential.

Despite the multiple advantages demonstrated in the design of RP14914P, this study has certain limitations. First, all results are based on bioinformatics predictions and require further validation through in vitro experiments, for instance, drawing on the ELISPOT method used for PP13138R or the cytokine detection scheme for PP19128R. N-methylpseudouridine modification will be incorporated during subsequent experimental synthesis to optimize the performance of the mRNA vaccine. Second, this study did not evaluate potential immune competition between epitopes or immunosuppressive effects mediated by regulatory T cells; this could be improved by referring to the analysis approach of T cell exhaustion markers used by Hu et al. [100]. Furthermore, the potential issue of overly strong immune reactions triggered by the dual TLR agonists may require further investigation. Finally, the optimization of the mRNA delivery system has not been addressed; strategies could explore the impact of delivery systems like lipid nanoparticles on vaccine efficacy, potentially drawing lessons from the antigen combination and delivery strategy of the B21 DNA vaccine by Weng et al. [101].

## 5. Conclusions

In this study, we designed and comprehensively evaluated RP14914P, a multi-epitope mRNA vaccine specifically targeting LTBI, using immunoinformatics and reverse vaccinology approaches. By integrating a three-step immunoinformatic screening pipeline, we selected 37 high-confidence T- and B-cell epitopes from seven well-characterized MTB latency-associated antigens. These epitopes were rationally assembled using immunologically optimized linkers and further reinforced with a built-in dual TLR2/4 agonist adjuvant system to enhance innate immune activation.

Computational structural analyses predicted that the vaccine exhibits a stable and soluble conformation, with 97.5% of residues in the most favored regions of the Ramachandran plot after refinement. Molecular docking and 100 ns MD simulations predicted strong and stable binding to TLR2 and TLR4, suggesting its potential to effectively engage pattern-recognition receptors. Importantly, conservation analysis revealed that the selected epitopes are highly conserved across 87 MTBC and NTM strains, with over 50% showing 100% sequence identity, indicating an indicator of potential broad-spectrum coverage.

In silico immune simulation projected robust and coordinated immune activation, characterized by Th1-polarized cytokine secretion (IFN-γ and IL-2), strong cytotoxic and helper T-cell responses, and durable B-cell memory. Together with a favorable safety profile, high predicted population coverage (≥82% for HLA-I and ≥99% for HLA-II), and optimized mRNA stability, RP14914P represents a rationally designed, computationally validated candidate that may specifically address the critical gap in preventing LTBI reactivation.

It is important to emphasize that all findings presented in this study are based on computational predictions and in silico analyses. While these results are promising and provide a strong rationale for further development, they require thorough experimental validation, including in vitro immunogenicity assays and in vivo challenge studies to confirm the vaccine’s safety, immunogenicity, and protective efficacy. This study provides a comprehensive immunoinformatic blueprint that can guide and accelerate subsequent experimental efforts toward developing a next-generation TB vaccine.

## Figures and Tables

**Table 1:** Biological Characteristics of Selected MTB Antigens and Their Potential in Vaccine Development and LTBI Differential Diagnosis.

Antigen	Type ^a^	Phase	Antigenicity ^b^	Allergenicity ^c^	Toxicity ^d^	Function	Functional Category	Vaccine Development Status	LTBI Differential Diagnosis	References
Rv1736c	DosR	Latent	0.4753	N	N	Involved in nitrate reduction, and in the persistence in the host	Intermediary metabolism and respiration	It performs well in preclinical trials and may be a good candidate vaccine	Induced by MTB rather than 13 BCG strains or *M. bovis*	[25]
Rv1980c	Other	Latent	0.6007	N	N	Detoxification of reactive oxygen species, contributes to drug resistance	Cell wall and cell processes	NA	Sensitivity and specificity were 0.92 and 0.95, respectively, sensitivity of the MPT64 test was significantly higher in TB-infected children than in adults.	[26]
Rv2656c	NS	Latent	0.5239	N	N	NA	Insertion seqs and phages	NA	NA	NA
Rv2659c	NS	Latent	0.5055	N	N	Sequence integration. Integrase is necessary for integration of a phage into the host genome by site-specific recombination	Insertion seqs and phages	rBCG Aure C::hly	Higher IFN-γ-producing T cells in LTBI vs. aTB and HC	[27]
Rv3879c	Other	Latent	0.6104	N	N	NA	Cell wall and cell processes	NA	The immunodominance of Rv3879c is higher than that of Rv3878 and Rv3873 in aTB and LTBI subjects.	[28]
Rv3872	Other	Latent	0.4894	N	N	NA	PE/PPE	NA	(1) Sensitivity and specificity of PE35 for detecting LTBI in children were 76% and80%. (2) Elicited stronger immunoreactivity and could discriminate TB from HC vaccinated with BCG (better than Rv3878).	[29,30]
Rv3873	NS	Latent	0.6381	N	N	lmmune modulation, interacts with humanTLR2, stimulates IL-10 and MCP-1 secretion	PE/PPE	H107e/CAF^®^10b	Sensitivity and specificity of PE68 for detecting LTBI in children were 73% and 75%.	[30]

Abbreviations: TLR, Toll-like receptors; IL-10, Interleukin-10; MCP-1, Monocyte Chemoattractant Protein-1; IFN-γ, interferon-gamma; ^a^ DosRs: dormancy survival regulon antigens; NS: nutrition starvation-associated antigens. ^b^ The antigenicity of proteins is evaluated using the VaxiJen v2.0 server, with a threshold value set at 0.45. ^c^ N indicates that the antigen has no known or discovered allergenic properties, meaning it is unlikely to cause allergic reactions. ^d^ N indicates that the antigen has no toxicity, or no toxicity has been discovered yet. NA: Not Available or Not Applicable.

**Table 2:** Immunoinformatic Characterization of CTL, HTL, and B-cell Epitopes Selected for the Multi-Epitope Vaccine RP149149P.

Protein	Peptide Sequence	Length	Alleles	Percentile Rank ^a^	IC50 ^b^	Antigenicity Score ^c^	IFN-γ Score ^d^	Immunogenicity Score ^e^	ABC Pred Score ^f^	IL4 ^g^	IL10 ^h^	AllerTOP V 2.0 ^i^	Toxin Pred ^j^
CTL epitopes													
Rv1736c	SPMFHFGIL	9	HLA-B*07:02/08:01	0.04	3.89	3.8900	NA	0.3089	NA	NA	NA	Non	Non
	FMATTVNDKV	10	HLA-A*02:03/02:01	0.28	4.27	4.2700	NA	0.0164	NA	NA	NA	Non	Non
	VVLEFAATV	9	HLA-A*02:06/02:01/02:03	0.06	7.85	7.8500	NA	0.3037	NA	NA	NA	Non	Non
Rv1980c	GGTHPTTTYK	10	HLA-A*11:01	0.02	21.79	1.6270	NA	0.1393	NA	NA	NA	Non	Non
Rv2656c	APTLAAAVEW	10	HLA-B*58:01	0.09	18.05	0.6194	NA	0.2078	NA	NA	NA	Non	Non
	YVAPTLAAAV	10	HLA-A*02:06	0.20	5.43	0.5868	NA	0.1213	NA	NA	NA	Non	Non
	DAARHWALR	9	HLA-A*68:01	0.15	9.92	0.5705	NA	0.3313	NA	NA	NA	Non	Non
Rv2659	IDLHGEVARV	10	HLA-A*02:03	0.24	20.23	1.4721	NA	0.2616	NA	NA	NA	Non	Non
	RTRAHYRKL	9	HLA-A*30:01	0.04	6.31	1.0337	NA	0.0019	NA	NA	NA	Non	Non
Rv3872	SQIDDGAAGV	10	HLA-A*02:06	0.21	21.71	1.0865	NA	0.2098	NA	NA	NA	Non	Non
Rv3873	MQATAQAAAY	10	HLA-B*15:01/35:01 HLA-A*30:02	0.04	4.88	0.5750	NA	0.0698	NA	NA	NA	Non	Non
	TEMDYFIRM	9	HLA-B*44:03	0.04	32.71	0.5697	NA	0.2145	NA	NA	NA	Non	Non
Rv3879	RDTRGREISA	10	HLA-A*30:01	0.36	28.91	2.5449	NA	0.2580	NA	NA	NA	Non	Non
	RYYANVTGRR	10	HLA-A*31:01	0.13	15.61	1.1056	NA	0.1675	NA	NA	NA	Non	Non
HTL epitopes													
Rv1736c	DPVAAWADIQADPRRRRR	18	HLA-DRB1*03:01/03:02/05:01 HLA-DQA1*01:01/03:01	0.47	NA	0.5475	1.6921	NA	NA	Non	Non	Non	Non
Rv1980c	NAGLDPVNYQNFAVTNDG	18	HLA-DPA1*03:01/DPB1*04:02 HLA-DPA1*01:03/DPB1*04:0 HLA-DPA1*02:01/DPB1*01:01 HLA-DPA1*01:03/DPB1*02:01 HLA-DRB1*04:05/15:01 HLA-DPA1*02:01/DPB1*05:01 HLA-DQA1*01:01/DQB1*05:01	0.57	NA	0.5137	0.4837	NA	NA	Non	Non	Non	Non
	SSTPREAPYELNITSATY	18	HLA-DRB3*02:02	0.44	NA	0.7701	0.5404	NA	NA	Non	Non	Non	Non
Rv2656c	APTLAAAVEWPMAGT	15	HLA-DRB1*09:01	0.45	NA	0.5853	0.0141	NA	NA	Non	Non	Non	Non
Rv2659c	AALRYQHAAKGR	12	HLA-DRB4*01:01	0.06	NA	1.0668	0.4385	NA	NA	Non	Non	Non	Non
Rv3872	SHDPIAADIGTQVSDNAL	18	HLA-DRB3*01:01	0.21	NA	0.5918	0.0183	NA	NA	Non	Non	Non	Non
Rv3873	QAKTRAMQATAQAAAYT	17	HLA-DQA1*01:02, HLA-DQB1*06:02	0.43	NA	0.7682	0.4548	NA	NA	Non	Non	Non	Non
Rv3879c	GLAPAIRNLADARLGVTL	18	HLA-DQA1*01:01, HLA-DQB1*05:01	0.47	NA	0.6025	0.4770	NA	NA	Non	Non	Non	Non
	LAPAIRNLADARLGVTL	17	HLA-DQA1*01:01, HLA-DQB1*05:01	0.48	NA	0.5405	0.1502	NA	NA	Non	Non	Non	Non
B cellular epitopes													
Rv1736c	HTISTYGPDRVAGFSP	16	NA	NA	NA	NA	NA	NA	0.94	NA	NA	Non	Non
	AVGSWWRYRYDKFGWT	16	NA	NA	NA	NA	NA	NA	0.93	NA	NA	Non	Non
Rv1980c	SIAPNAGLDPVNYQNF	16	NA	NA	NA	NA	NA	NA	0.92	NA	NA	Non	Non
	AATSSTPREAPYELNI	16	NA	NA	NA	NA	NA	NA	0.92	NA	NA	Non	Non
Rv2656c	TPSSTDPTASRAVSWW	16	NA	NA	NA	NA	NA	NA	0.93	NA	NA	Non	Non
	SREIQRRRDAYIRRVV	16	NA	NA	NA	NA	NA	NA	0.81	NA	NA	Non	Non
Rv2659c	GRVYIAPKTFNAKIDA	16	NA	NA	NA	NA	NA	NA	0.97	NA	NA	Non	Non
	ATTAVGTPTMRAHSYS	16	NA	NA	NA	NA	NA	NA	0.9	NA	NA	Non	Non
Rv3872	EKMSHDPIAADIGTQV	16	NA	NA	NA	NA	NA	NA	0.87	NA	NA	Non	Non
	MTERCLSISHRVRVPE	16	NA	NA	NA	NA	NA	NA	0.84	NA	NA	Non	Non
Rv3873	APLAQEREEDDEDDWD	16	NA	NA	NA	NA	NA	NA	0.93	NA	NA	Non	Non
	SSTPVGQLPPAATQTL	16	NA	NA	NA	NA	NA	NA	0.85	NA	NA	Non	Non
Rv3879c	LIPLNPTPGSDWDASP	16	NA	NA	NA	NA	NA	NA	0.86	NA	NA	Non	Non
	APAIRNLADARLGVTL	16	NA	NA	NA	NA	NA	NA	0.84	NA	NA	Non	Non

Abbreviations: HTL, helper T lymphocyte; CTL, cytotoxic T lymphocyte; IFN-γ, interferon-gamma; IC50, half maximal inhibitory concentration; IL, interleukin. ^a^ The percentile ranking of the selected epitopes, and the inclusion criterion is the ranking score less than 0.5. ^b^ The half maximal inhibitory concentration test, epitopes with IC50 value < 50 nM were selected. ^c^ The antigenicity score, epitopes with antigenicity score greater than 0.5 were selected. ^d^ IFN-γ score, The epitope with a positive score and the highest score is selected. ^e^ The immunogenicity score, epitopes were selected in order of score. ^f^ The linear B-cell epitope prediction score, epitopes were selected in order of score. ^g^ Stimulated cytokine IL-4 secretion, HTL epitopes that do not secrete cytokine IL-4 were selected. ^h^ Stimulated cytokine IL-10 secretion, HTL epitopes that do not secrete cytokine IL-10 were selected. ^i^ The result of the sensitization test, “Non” indicates that the epitope was non-sensitizing. ^j^ The result of the Toxin, “Non” indicates that the epitope was non-toxic. NA: Not Available or Not Applicable.

## Data Availability

The original contributions presented in this study are included in the article/Supplementary Material. Further inquiries can be directed to the corresponding author.

## Supplementary Materials

The following supporting information can be downloaded at: https://www.mdpi.com/article/10.3390/pathogens15030297/s1, Table S1: Preliminary screening results of cytotoxic T-lymphocyte (CTL) epitopes; Table S2: Preliminary screening results of helper T-lymphocyte (HTL) epitopes; Table S3: Preliminary screening results of linear B-cell epitopes; Table S4: Analysis of Physicochemical Properties and MHC Coverage of Antigens with Different Topological Scaffolds; Table S5: Vaccines and Their Physicochemical Properties During Agonist Screening; Table S6: Structural Scores of the RP14914P Vaccine Model After Optimization; Table S7: Discontinuous B-cell epitopes in the RP14914P structure predicted by ElliPro.

## Abbreviations

ATB	Active Tuberculosis
BCG	Bacillus Calmette-Guérin
CTL	Cytotoxic T Lymphocyte
DCCM	Dynamic Cross-Correlation Matrix
HLA	Human Leukocyte Antigen
HTL	Helper T Lymphocyte
IFN-γ	Interferon-Gamma
IL-2	Interleukin-2
IL-4	Interleukin-4
IL-10	Interleukin-10
LNP	Lipid Nanoparticle
LTBI	Latent Tuberculosis Infection
MHC	Major Histocompatibility Complex
MTB	*Mycobacterium Tuberculosis*
MTBC	*Mycobacterium Tuberculosis* Complex
Rg	Radius of Gyration
RMSD	Root Mean Square Deviation
RMSF	Root Mean Square Fluctuation
TC	Cytotoxic T Cell
TH	Helper T Cell
TGF-β	Transforming Growth Factor-Beta
TLR	Toll-Like Receptor
WHO	World Health Organization
NK Cell	Natural Killer Cell

## References

[1] Liu Y., Yang L., Meskini M., Goel A., Opperman M., Shyamal S.S., Manaithiya A., Xiao M., Ni R., An Y. (2025). Gut microbiota and tuberculosis. IMeta.

[2] Chen Z., Wang T., Du J., Sun L., Wang G., Ni R., An Y., Fan X., Li Y., Guo R. (2025). Decoding the WHO Global Tuberculosis Report 2024: A Critical Analysis of Global and Chinese Key Data. Zoonoses.

[3] An Y., Ni R., Zhuang L., Yang L., Ye Z., Li L., Parkkila S., Aspatwar A., Gong W. (2025). Tuberculosis vaccines and therapeutic drug: Challenges and future directions. Mol. Biomed..

[4] Hatzenbuehler L.A., Starke J.R. (2014). Current Diagnosis and Treatment of Pediatric Latent Tuberculosis Infection. Curr. Pediatr. Rep..

[5] Doshi J., Deokar K., Gaikwad P. (2024). Novel tuberculosis skin tests for detecting latent tuberculosis infection. Monaldi Arch. Chest Dis..

[6] Chee C.B.E., Reves R., Zhang Y., Belknap R. (2018). Latent tuberculosis infection: Opportunities and challenges. Respirology.

[7] Carranza C., Pedraza-Sanchez S., de Oyarzabal-Mendez E., Torres M. (2020). Diagnosis for Latent Tuberculosis Infection: New Alternatives. Front. Immunol..

[8] WHO (2025). Global Tuberculosis Report 2025.

[9] Gong W., Wu X. (2021). Differential Diagnosis of Latent Tuberculosis Infection and Active Tuberculosis: A Key to a Successful Tuberculosis Control Strategy. Front. Microbiol..

[10] Mangtani P., Abubakar I., Ariti C., Beynon R., Pimpin L., Fine P.E., Rodrigues L.C., Smith P.G., Lipman M., Whiting P.F. (2014). Protection by BCG vaccine against tuberculosis: A systematic review of randomized controlled trials. Clin. Infect. Dis..

[11] Zhuang L., Ye Z., Li L., Yang L., Gong W. (2023). Next-Generation TB Vaccines: Progress, Challenges, and Prospects. Vaccines.

[12] Tait D.R., Hatherill M., Van Der Meeren O., Ginsberg A.M., Van Brakel E., Salaun B., Scriba T.J., Akite E.J., Ayles H.M., Bollaerts A. (2019). Final Analysis of a Trial of M72/AS01(E) Vaccine to Prevent Tuberculosis. N. Engl. J. Med..

[13] Luabeya A.K., Kagina B.M., Tameris M.D., Geldenhuys H., Hoff S.T., Shi Z., Kromann I., Hatherill M., Mahomed H., Hanekom W.A. (2015). First-in-human trial of the post-exposure tuberculosis vaccine H56:IC31 in Mycobacterium tuberculosis infected and non-infected healthy adults. Vaccine.

[14] Aagaard C., Hoang T., Dietrich J., Cardona P.J., Izzo A., Dolganov G., Schoolnik G.K., Cassidy J.P., Billeskov R., Andersen P. (2011). A multistage tuberculosis vaccine that confers efficient protection before and after exposure. Nat. Med..

[15] O’Garra A., Redford P.S., McNab F.W., Bloom C.I., Wilkinson R.J., Berry M.P. (2013). The immune response in tuberculosis. Annu. Rev. Immunol..

[16] Rezk N., McClean S. (2025). Harnessing the Potential of mRNA Vaccines Against Infectious Diseases. Microb. Biotechnol..

[17] Sethi G., Kim Y.K., Han S.C., Hwang J.H. (2024). Designing a broad-spectrum multi-epitope subunit vaccine against leptospirosis using immunoinformatics and structural approaches. Front. Immunol..

[18] Yazdani Z., Rafiei A., Irannejad H., Yazdani M., Valadan R. (2022). Designing a novel multiepitope peptide vaccine against melanoma using immunoinformatics approach. J. Biomol. Struct. Dyn..

[19] Tan C., Xiao Y., Liu T., Chen S., Zhou J., Zhang S., Hu Y., Wu A., Li C. (2024). Development of multi-epitope mRNA vaccine against Clostridioides difficile using reverse vaccinology and immunoinformatics approaches. Synth. Syst. Biotechnol..

[20] Ramprasadh S.V., Rajakumar S., Srinivasan S., Susha D., Sharma S., Chourasiya R. (2023). Computer-Aided Multi-Epitope Based Vaccine Design Against Monkeypox Virus Surface Protein A30L: An Immunoinformatics Approach. Protein J..

[21] Fathollahi M., Motamedi H., Hossainpour H., Abiri R., Shahlaei M., Moradi S., Dashtbin S., Moradi J., Alvandi A. (2023). Designing a novel multi-epitopes pan-vaccine against SARS-CoV-2 and seasonal influenza: In silico and immunoinformatics approach. J. Biomol. Struct. Dyn..

[22] Zhang M., Ali S.L., Tian Y., Abduldayeva A., Zhou S., An Y., Li Y., Ni R., Zhang L., Liu Y. (2025). EP9158H: An Immunoinformatics-Designed mRNA Vaccine Encoding Multi-Epitope Antigens and Dual TLR Agonists for Tuberculosis Prevention. Bioengineering.

[23] Shahrear S., Islam A.B.M.M.K. (2022). Modeling of MT. P495, an mRNA-based vaccine against the phosphate-binding protein PstS1 of Mycobacterium tuberculosis. Mol. Divers..

[24] Xu S., Yang K., Li R., Zhang L. (2020). mRNA Vaccine Era-Mechanisms, Drug Platform and Clinical Prospection. Int. J. Mol. Sci..

[25] Honaker R.W., Stewart A., Schittone S., Izzo A., Klein M.R., Voskuil M.I. (2008). Mycobacterium bovis BCG vaccine strains lack narK2 and narX induction and exhibit altered phenotypes during dormancy. Infect. Immun..

[26] Cao X.J., Li Y.P., Wang J.Y., Zhou J., Guo X.G. (2021). MPT64 assays for the rapid detection of Mycobacterium tuberculosis. BMC Infect. Dis..

[27] Bai X. (2014). Preparation of Four Tuberculosis Latent Proteins and the Evaluation of Their Immunological Characteristic. Doctoral Dissertation.

[28] Hinks T.S., Dosanjh D.P., Innes J.A., Pasvol G., Hackforth S., Varia H., Millington K.A., Liu X.Q., Bakir M., Soysal A. (2009). Frequencies of region of difference 1 antigen-specific but not purified protein derivative-specific gamma interferon-secreting T cells correlate with the presence of tuberculosis disease but do not distinguish recent from remote latent infections. Infect. Immun..

[29] Mukherjee P., Dutta M., Datta P., Dasgupta A., Pradhan R., Pradhan M., Kundu M., Basu J., Chakrabarti P. (2007). The RD1-encoded antigen Rv3872 of Mycobacterium tuberculosis as a potential candidate for serodiagnosis of tuberculosis. Clin. Microbiol. Infect..

[30] Mahmoudi S., Pourakbari B., Mamishi S. (2017). Interferon Gamma Release Assay in response to PE35/PPE68 proteins: A promising diagnostic method for diagnosis of latent tuberculosis. Eur. Cytokine Netw..

[31] Reynisson B., Alvarez B., Paul S., Peters B., Nielsen M. (2020). NetMHCpan-4.1 and NetMHCIIpan-4.0: Improved predictions of MHC antigen presentation by concurrent motif deconvolution and integration of MS MHC eluted ligand data. Nucleic Acids Res..

[32] Kaabinejadian S., Barra C., Alvarez B., Yari H., Hildebrand W.H., Nielsen M. (2022). Accurate MHC Motif Deconvolution of Immunopeptidomics Data Reveals a Significant Contribution of DRB3, 4 and 5 to the Total DR Immunopeptidome. Front. Immunol..

[33] Arif S., Aslam F. (2025). Immunoinformatics-driven construction of a next-generation epitope-based vaccine from conserved hypothetical proteins of M. tuberculosis for enhanced TB control. Comput. Biol. Med..

[34] Dhanda S.K., Gupta S., Vir P., Raghava G.P. (2013). Prediction of IL4 inducing peptides. Clin. Dev. Immunol..

[35] Nagpal G., Usmani S.S., Dhanda S.K., Kaur H., Singh S., Sharma M., Raghava G.P. (2017). Computer-aided designing of immunosuppressive peptides based on IL-10 inducing potential. Sci. Rep..

[36] Saha S., Raghava G.P. (2006). Prediction of continuous B-cell epitopes in an antigen using recurrent neural network. Proteins.

[37] Doytchinova I.A., Flower D.R. (2007). VaxiJen: A server for prediction of protective antigens, tumour antigens and subunit vaccines. BMC Bioinform..

[38] Dimitrov I., Bangov I., Flower D.R., Doytchinova I. (2014). AllerTOP v.2—A server for in silico prediction of allergens. J. Mol. Model..

[39] Sharma N., Naorem L.D., Jain S., Raghava G.P.S. (2022). ToxinPred2: An improved method for predicting toxicity of proteins. Brief. Bioinform..

[40] Shi H., Zhu Y., Shang K., Tian T., Yin Z., Shi J., He Y., Ding J., Wang Q., Zhang F. (2024). Development of innovative multi-epitope mRNA vaccine against central nervous system tuberculosis using in silico approaches. PLoS ONE.

[41] Reshetnikov V., Terenin I., Shepelkova G., Yeremeev V., Kolmykov S., Nagornykh M., Kolosova E., Sokolova T., Zaborova O., Kukushkin I. (2024). Untranslated Region Sequences and the Efficacy of mRNA Vaccines against Tuberculosis. Int. J. Mol. Sci..

[42] Saadh M.J., Muhammad F.A., Albadr R.J., Ballal S., Singh A., Devi A., Joshi K.K., Saidkhodjaeva S., Taher W.M., Alwan M. (2025). Structure-based design of a multi-epitope vaccine candidate against marburg virus using immunoinformatics and dynamics simulations. J. Mol. Graph. Model..

[43] Hebditch M., Carballo-Amador M.A., Charonis S., Curtis R., Warwicker J. (2017). Protein-Sol: A web tool for predicting protein solubility from sequence. Bioinformatics.

[44] Barman A., Deb B., Chakraborty S. (2020). Prediction of potential epitopes for peptide vaccine formulation against teschovirus a using immunoinformatics. Int. J. Pept. Res. Ther..

[45] Long S., Tian P. (2019). Protein secondary structure prediction with context convolutional neural network. RSC Adv..

[46] McGuffin L.J., Bryson K., Jones D.T. (2000). The PSIPRED protein structure prediction server. Bioinformatics.

[47] Haron F.N., Azazi A., Chua K.H., Lim Y.A.L., Lee P.C., Chew C.H. (2022). In silico structural modeling and quality assessment of Plasmodium knowlesi apical membrane antigen 1 using comparative protein models. Trop. Biomed..

[48] Muccee F., Ghazanfar S., Ajmal W., Al-Zahrani M. (2022). In-silico characterization of estrogen reactivating β-glucuronidase enzyme in git associated microbiota of normal human and breast cancer patients. Genes.

[49] Gong W., Liang Y., Mi J., Jia Z., Xue Y., Wang J., Wang L., Zhou Y., Sun S., Wu X. (2021). Peptides-Based Vaccine MP3RT Induced Protective Immunity Against Mycobacterium Tuberculosis Infection in a Humanized Mouse Model. Front. Immunol..

[50] Gong W., Liang Y., Mi J., Xue Y., Wang J., Wang L., Zhou Y., Sun S., Wu X. (2022). A peptide-based vaccine ACP derived from antigens of *Mycobacterium tuberculosis* induced Th1 response but failed to enhance the protective efficacy of BCG in mice. Indian J. Tuberc..

[51] Setlur A.S., K C., Pandey S., Sarkar M., Niranjan V. (2023). Comprehensive Molecular Interaction Studies to Construe the Repellent/Kill Activity of Geraniol During Binding Event Against Aedes aegypti Proteins. Mol. Biotechnol..

[52] Bui H.H., Sidney J., Dinh K., Southwood S., Newman M.J., Sette A. (2006). Predicting population coverage of T-cell epitope-based diagnostics and vaccines. BMC Bioinform..

[53] Ponomarenko J., Bui H.H., Li W., Fusseder N., Bourne P.E., Sette A., Peters B. (2008). ElliPro: A new structure-based tool for the prediction of antibody epitopes. BMC Bioinform..

[54] Jones G., Jindal A., Ghani U., Kotelnikov S., Egbert M., Hashemi N., Vajda S., Padhorny D., Kozakov D. (2022). Elucidation of protein function using computational docking and hotspot analysis by ClusPro and FTMap. Acta Crystallogr. D Struct. Biol..

[55] Ullah A., Rehman B., Khan S., Almanaa T.N., Waheed Y., Hassan M., Naz T., Ul Haq M., Muhammad R., Sanami S. (2024). An In Silico Multi-epitopes Vaccine Ensemble and Characterization Against Nosocomial Proteus penneri. Mol. Biotechnol..

[56] Zhang M., Wei N., Lin R., Xu Y., Zhang Q., Jia L., Zhang X., Yang X. (2025). Deferoxamine addresses metabolic dysregulation and urinary tract infections in weight-associated gestational diabetes mellitus. Eur. J. Med. Res..

[57] Lopes C.M.R., de Araújo L.P., Mariano C.P., Falleiros L., de Azevedo Junior W.F., Coelho L.F.L., da Silveira N.J.F. (2025). Bioisosteric and Virtual Screening Approach to Identify Natural Inhibitors of Chikunguya alphavirus nsP3. Cell Biochem. Biophys..

[58] Singh A., Pandit S., Singh T.P., Sharma S., Sharma P. (2025). Disruption of histidine biosynthesis in Acinetobacter baumannii by Tubuloside B from Cistanche tubulosa. Comput. Biol. Med..

[59] Rapin N., Lund O., Bernaschi M., Castiglione F. (2010). Computational immunology meets bioinformatics: The use of prediction tools for molecular binding in the simulation of the immune system. PLoS ONE.

[60] Zaib S., Rana N., Areeba, Hussain N., Alrbyawi H., Dera A.A., Khan I., Khalid M., Khan A., Al-Harrasi A. (2023). Designing multi-epitope monkeypox virus-specific vaccine using immunoinformatics approach. J. Infect. Public Health.

[61] Zuker M., Stiegler P. (1981). Optimal computer folding of large RNA sequences using thermodynamics and auxiliary information. Nucleic Acids Res..

[62] Salaikumaran M.R., Kasamuthu P.S., Aathmanathan V.S., Burra V. (2022). An in silico approach to study the role of epitope order in the multi-epitope-based peptide (MEBP) vaccine design. Sci. Rep..

[63] Gonzalez M.C., Kostrzak A., Guetard D., Pniewski T., Sala M. (2009). HIV-1 derived peptides fused to HBsAg affect its immunogenicity. Virus Res..

[64] Yang Y., Xue Y., Wang X., Wang L., Wang J., Zhang J., Liu Y., Liang Y., Wu X. (2024). Bioinformatics Analysis and Immunogenicity Assessment of the Novel Multi-Stage DNA Vaccine W541 Against Mycobacterium Tuberculosis. Immun. Inflamm. Dis..

[65] Tien N.T.N., Yen N.T.H., Phat N.K., Anh N.K., Thu N.Q., Eunsu C., Kim H.S., Hoa V.D., Nguyen D.N., Kim D.H. (2025). Multiomics and Machine Learning Identify Immunometabolic Biomarkers for Active Tuberculosis Diagnosis Against Nontuberculous Mycobacteria and Latent Tuberculosis Infection. J. Proteome Res..

[66] Bongomin F., Pitua I., Ssekamatte P., Sitenda D., Andia-Biraro I., Jonani B. (2025). Agreement and systematic bias between QuantiFERON–chemiluminescent immunoassay and QuantiFERON–enzyme-linked immunosorbent assay in the detection of latent tuberculosis infection: A systematic review and meta-analysis. IJID Reg..

[67] Zhang Z., Wang Y., Zhang Y., Geng S., Wu H., Shao Y., Kang G. (2024). Construction of Immune-Related Diagnostic Model for Latent Tuberculosis Infection and Active Tuberculosis. J. Inflamm. Res..

[68] Li F., Dang W., Du Y., Xu X., He P., Zhou Y., Zhu B. (2024). Tuberculosis Vaccines and T Cell Immune Memory. Vaccines.

[69] Moradi M., Vahedi F., Abbassioun A., Ramezanpour Shahi A., Sholeh M., Taheri-Anganeh M., Dargahi Z., Ghanavati R., Khatami S.H., Movahedpour A. (2023). Liposomal delivery system/adjuvant for tuberculosis vaccine. Immun. Inflamm. Dis..

[70] Schrager L.K., Vekemens J., Drager N., Lewinsohn D.M., Olesen O.F. (2020). The status of tuberculosis vaccine development. Lancet Infect. Dis..

[71] Kazakova A., Zhelnov P., Sidorov R., Rogova A., Vasileva O., Ivanov R., Reshetnikov V., Muslimov A. (2024). DNA and RNA vaccines against tuberculosis: A scoping review of human and animal studies. Front. Immunol..

[72] Kozlova A., Pateev I., Shepelkova G., Vasileva O., Zakharova N., Yeremeev V., Ivanov R., Reshetnikov V. (2024). A Cap-Optimized mRNA Encoding Multiepitope Antigen ESAT6 Induces Robust Cellular and Humoral Immune Responses Against Mycobacterium tuberculosis. Vaccines.

[73] Xue T., Stavropoulos E., Yang M., Ragno S., Vordermeier M., Chambers M., Hewinson G., Lowrie D.B., Colston M.J., Tascon R.E. (2004). RNA encoding the MPT83 antigen induces protective immune responses against Mycobacterium tuberculosis infection. Infect. Immun..

[74] Lorenzi J.C., Trombone A.P., Rocha C.D., Almeida L.P., Lousada R.L., Malardo T., Fontoura I.C., Rossetti R.A., Gembre A.F., Silva A.M. (2010). Intranasal vaccination with messenger RNA as a new approach in gene therapy: Use against tuberculosis. BMC Biotechnol..

[75] Chugh S., Bahal R.K., Dhiman R., Singh R. (2024). Antigen identification strategies and preclinical evaluation models for advancing tuberculosis vaccine development. npj Vaccines.

[76] Agrawal N., Ates L.S., Schille S., Chaturvedi A., Vogt J., Vukovic N., Vogel A.B., Diekmann J., Diken M., Şahin U. (2025). mRNA-based tuberculosis vaccines BNT164a1 and BNT164b1 are immunogenic, well-tolerated and efficacious in rodent models. bioRxiv.

[77] Jiang F., Peng C., Cheng P., Wang J., Lian J., Gong W. (2023). PP19128R, a Multiepitope Vaccine Designed to Prevent Latent Tuberculosis Infection, Induced Immune Responses In Silico and In Vitro Assays. Vaccines.

[78] Al Tbeishat H. (2022). Novel In Silico mRNA vaccine design exploiting proteins of M. tuberculosis that modulates host immune responses by inducing epigenetic modifications. Sci. Rep..

[79] Zhuang L., Ali A., Yang L., Ye Z., Li L., Ni R., An Y., Ali S.L., Gong W. (2024). Leveraging computer-aided design and artificial intelligence to develop a next-generation multi-epitope tuberculosis vaccine candidate. Infect. Med..

[80] Kazakova A.A., Shepelkova G.S., Kukushkin I.S., Yeremeev V.V., Ivanov R.A., Reshetnikov V.V. (2025). Multiepitope mRNA Vaccine mRNA-mEp21-FL-IDT Provides Efficient Protection against *M. tuberculosis*. Biochemistry.

[81] Nell A.S., D’Lom E., Bouic P., Sabaté M., Bosser R., Picas J., Amat M., Churchyard G., Cardona P.J. (2014). Safety, tolerability, and immunogenicity of the novel antituberculous vaccine RUTI: Randomized, placebo-controlled phase II clinical trial in patients with latent tuberculosis infection. PLoS ONE.

[82] Correia B.E., Bates J.T., Loomis R.J., Baneyx G., Carrico C., Jardine J.G., Rupert P., Correnti C., Kalyuzhniy O., Vittal V. (2014). Proof of principle for epitope-focused vaccine design. Nature.

[83] Tahir Ul Qamar M., Saleem S., Ashfaq U.A., Bari A., Anwar F., Alqahtani S. (2019). Epitope-based peptide vaccine design and target site depiction against Middle East Respiratory Syndrome Coronavirus: An immune-informatics study. J. Transl. Med..

[84] Sela-Culang I., Kunik V., Ofran Y. (2013). The structural basis of antibody-antigen recognition. Front. Immunol..

[85] Cheng P., Jiang F., Wang G., Wang J., Xue Y., Wang L., Gong W. (2023). Bioinformatics analysis and consistency verification of a novel tuberculosis vaccine candidate HP13138PB. Front. Immunol..

[86] Tan Y., Kagan J.C. (2014). A cross-disciplinary perspective on the innate immune responses to bacterial lipopolysaccharide. Mol. Cell.

[87] Nussinov R., Tsai C.J., Jang H. (2021). Anticancer drug resistance: An update and perspective. Drug Resist. Updat..

[88] Ghisletti S., Barozzi I., Mietton F., Polletti S., De Santa F., Venturini E., Gregory L., Lonie L., Chew A., Wei C.L. (2010). Identification and characterization of enhancers controlling the inflammatory gene expression program in macrophages. Immunity.

[89] Blum J.S., Wearsch P.A., Cresswell P. (2013). Pathways of antigen processing. Annu. Rev. Immunol..

[90] Zhu J., Yamane H., Paul W.E. (2010). Differentiation of effector CD4 T cell populations (*). Annu. Rev. Immunol..

[91] Schoenborn J.R., Wilson C.B. (2007). Regulation of interferon-gamma during innate and adaptive immune responses. Adv. Immunol..

[92] Halle S., Keyser K.A., Stahl F.R., Busche A., Marquardt A., Zheng X., Galla M., Heissmeyer V., Heller K., Boelter J. (2016). In Vivo Killing Capacity of Cytotoxic T Cells Is Limited and Involves Dynamic Interactions and T Cell Cooperativity. Immunity.

[93] Kaech S.M., Cui W. (2012). Transcriptional control of effector and memory CD8+ T cell differentiation. Nat. Rev. Immunol..

[94] Badovinac V.P., Harty J.T. (2006). Programming, demarcating, and manipulating CD8+ T-cell memory. Immunol. Rev..

[95] Pape K.A., Taylor J.J., Maul R.W., Gearhart P.J., Jenkins M.K. (2011). Different B cell populations mediate early and late memory during an endogenous immune response. Science.

[96] Plotkin S.A. (2010). Correlates of protection induced by vaccination. Clin. Vaccine Immunol..

[97] Larsen S.E., Erasmus J.H., Reese V.A., Pecor T., Archer J., Kandahar A., Hsu F.C., Nicholes K., Reed S.G., Baldwin S.L. (2023). An RNA-Based Vaccine Platform for Use against Mycobacterium tuberculosis. Vaccines.

[98] Rais M., Abdelaal H., Reese V.A., Ferede D., Larsen S.E., Pecor T., Erasmus J.H., Archer J., Khandhar A.P., Cooper S.K. (2023). Immunogenicity and protection against Mycobacterium avium with a heterologous RNA prime and protein boost vaccine regimen. Tuberculosis.

[99] Jiang F., Han Y., Liu Y., Xue Y., Cheng P., Xiao L., Gong W. (2023). A comprehensive approach to developing a multi-epitope vaccine against Mycobacterium tuberculosis: From in silico design to in vitro immunization evaluation. Front. Immunol..

[100] Hu Z., Xia J., Wu J., Zhao H., Ji P., Gu L., Gu W., Chen Z., Xu J., Huang X. (2024). A multistage Sendai virus vaccine incorporating latency-associated antigens induces protection against acute and latent tuberculosis. Emerg. Microbes Infect..

[101] Weng S., Zhang J., Ma H., Zhou J., Jia L., Wan Y., Cui P., Ruan Q., Shao L., Wu J. (2022). B21 DNA vaccine expressing ag85b, rv2029c, and rv1738 confers a robust therapeutic effect against latent Mycobacterium tuberculosis infection. Front. Immunol..

