# Supplementary material for: Immunoinformatic Design and Evaluation of a Multi-Epitope mRNA Vaccine RP14914P Targeting Latent Tuberculosis Infection
Source: Pathogens. 2026 Mar 9;15(3):297. doi: 10.3390/pathogens15030297 (PMC13029540; doi:10.3390/pathogens15030297)
Supplement: Supplementary file 1 [file pathogens-15-00297-s001.zip › pathogens-4066419-supplementary.pdf]

Supplementary Materials

Table S1. Preliminary screening results of cytotoxic T-lymphocyte (CTL) epitopes.

Antigen	peptide	start	end	peptide length	allele	netmhcpan_el percentile	netmhcpan_ba IC50	immunogenicity score	Antigenicity	Allergenicity	Toxicity
RV1736C	IGLALFALW	605	613	9	HLA-B*58:01	0.18	10.54	0.16171	1.5904	NON-ALLERGEN	Non-Toxin
RV1736C	WTAGDTRNR	328	336	9	HLA-A*68:01	0.19	21.48	0.14484	1.4316	NON-ALLERGEN	Non-Toxin
RV1736C	ELFWTVVPYV	419	428	10	HLA-A*68:02	0.78	26.87	0.36623	1.27	NON-ALLERGEN	Non-Toxin
RV1736C	RTVTRRGGR	32	40	9	HLA-A*31:01	0.2	14.06	0.19998	1.2205	NON-ALLERGEN	Non-Toxin
RV1736C	FLPVVLEFA	362	370	9	HLA-A*02:03	0.48	12.16	0.2206	1.1156	NON-ALLERGEN	Non-Toxin
RV1736C	MYLVLVAAI	543	551	9	HLA-A*23:01	0.33	30.78	0.12188	0.9414	NON-ALLERGEN	Non-Toxin
RV1736C	SPMFHFGIL	465	473	9	HLA-B*07:02	0.04	3.89	0.30886	0.9368	NON-ALLERGEN	Non-Toxin
RV1736C	FMATTVNDKV	533	542	10	HLA-A*02:03	0.28	4.27	0.01639	0.7952	NON-ALLERGEN	Non-Toxin
RV1736C	VVLEFAATV	365	373	9	HLA-A*02:06	0.06	7.85	0.30368	0.7487	NON-ALLERGEN	Non-Toxin
RV1736C	RYDKFGWTTR	442	451	10	HLA-A*31:01	0.1	12.18	0.20115	0.6887	NON-ALLERGEN	Non-Toxin
RV1736C	RSTDVAPPR	348	356	9	HLA-A*31:01	0.08	28.63	0.09611	0.6798	NON-ALLERGEN	Non-Toxin
RV1736C	GAAFSWYTY	106	114	9	HLA-A*30:02	0.16	31.16	0.19747	0.6449	NON-ALLERGEN	Non-Toxin
RV1736C	SELRRADPL	297	305	9	HLA-B*40:01	0.18	37.33	0.14795	0.5775	NON-ALLERGEN	Non-Toxin
RV1736C	ISADLRTVTR	27	36	10	HLA-A*68:01	0.23	30.29	0.16868	0.5703	NON-ALLERGEN	Non-Toxin
RV1736C	YQIHVLIGL	599	607	9	HLA-A*02:06	0.03	2.92	0.23763	0.5163	NON-ALLERGEN	Non-Toxin
RV1980C	GGTHPTTTYK	121	130	10	HLA-A*11:01	0.02	21.79	0.13929	1.627	NON-ALLERGEN	Non-Toxin
RV2656C	APTLAAAVEW	48	57	10	HLA-B*58:01	0.09	18.05	0.20781	0.6194	NON-ALLERGEN	Non-Toxin
RV2656C	YVAPTLAAAV	46	55	10	HLA-A*02:06	0.2	5.43	0.12125	0.5868	NON-ALLERGEN	Non-Toxin
RV2656C	DAARHWALR	81	89	9	HLA-A*68:01	0.15	9.92	0.33133	0.5705	NON-ALLERGEN	Non-Toxin
RV2659	IDLHGEVARV	221	230	10	HLA-A*02:03	0.24	20.23	0.26157	1.4721	NON-ALLERGEN	Non-Toxin
RV2659	RTRAHYRKL	93	101	9	HLA-A*30:01	0.04	6.31	0.00187	1.0337	NON-ALLERGEN	Non-Toxin
RV2659	ATFADTDLR	108	116	9	HLA-A*68:01	0.16	12.5	0.14775	0.9854	NON-ALLERGEN	Non-Toxin
RV2659	KIRPATLDEL	176	185	10	HLA-A*30:01	0.18	44.4	0.14832	0.811	NON-ALLERGEN	Non-Toxin
RV3879	RDTRGREISA	170	179	10	HLA-A*30:01	0.36	28.91	0.25799	2.5449	NON-ALLERGEN	Non-Toxin
RV3879	RYYANVTGRR	88	97	10	HLA-A*31:01	0.13	15.61	0.16745	1.1056	NON-ALLERGEN	Non-Toxin
RV3872	SQIDDGAAGV	87	96	10	HLA-A*02:06	0.21	21.71	0.20977	1.0865	NON-ALLERGEN	Non-Toxin
RV3873	DAQAVELTAR	39	48	10	HLA-A*68:01	0.34	49.41	0.18465	1.3893	NON-ALLERGEN	Non-Toxin
RV3873	RPGLVAPAPL	341	350	10	HLA-B*07:02	0.1	9.41	0.09424	1.0552	NON-ALLERGEN	Non-Toxin
RV3873	AETAVNTLF	156	164	9	HLA-B*44:03	0.01	16.18	0.11236	0.5896	NON-ALLERGEN	Non-Toxin
RV3873	MQATAQAAAY	87	96	10	HLA-B*15:01	0.04	4.88	0.06977	0.575	NON-ALLERGEN	Non-Toxin
RV3873	TEMDYFIRM	135	143	9	HLA-B*44:03	0.04	32.71	0.21448	0.5697	NON-ALLERGEN	Non-Toxin

Table S2. Preliminary screening results of helper T-lymphocyte (HTL) epitopes.

Antigen	allele	seq_num	start	end	length	core_peptide	peptide	score	rank	Antigenicity	Allergenicity	Toxicity	IFN	IF10	IF4
RV1736C	HLA-DRB1*03:01	1	144	161	18	IQADPRRRR	AAWADIQADPRRRRRYQR	0.9248	0.15	0.7584	NON-ALLERGEN	Non-Toxin	0.99874106	IL10 non-inducer	Non IL4 inducer
RV1736C	HLA-DRB1*03:01	1	147	158	12	IQADPRRRR	ADIQADPRRRRR	0.9262	0.04	1.2588	NON-ALLERGEN	Non-Toxin	0.58767228	IL10 non-inducer	Non IL4 inducer
RV1736C	HLA-DRB1*03:01	1	145	162	18	IQADPRRRR	AWADIQADPRRRRRYQRA	0.9121	0.2	0.7818	NON-ALLERGEN	Non-Toxin	1.1049788	IL10 non-inducer	Non IL4 inducer
RV1736C	HLA-DRB1*03:01	1	141	158	18	IQADPRRRR	DPVAAWADIQADPRRRRR	0.8526	0.47	0.5475	NON-ALLERGEN	Non-Toxin	1.692108	IL10 non-inducer	Non IL4 inducer
RV1736C	HLA-DRB1*03:01	1	142	159	18	IQADPRRRR	PVAAWADIQADPRRRRRY	0.9016	0.25	0.6244	NON-ALLERGEN	Non-Toxin	1.0862821	IL10 non-inducer	Non IL4 inducer
RV1980C	HLA-DPA1*03:01/DPB1*04:02	1	179	196	18	VNYQNFAVT	NAGLDPVNYQNFAVTNDG	0.2873	0.57	0.5137	NON-ALLERGEN	Non-Toxin	0.48371364	IL10 non-inducer	Non IL4 inducer
RV1980C	HLA-DRB3*02:02	1	83	100	18	YELNITSAT	SSTPREAPYELNITSATY	0.3567	0.44	0.7701	NON-ALLERGEN	Non-Toxin	0.54040989	IL10 non-inducer	Non IL4 inducer
RV2656C	HLA-DRB1*09:01	1	48	62	15	LAAAVEWPM	APTLAAAVEWPMAGT	0.7567	0.45	0.5853	NON-ALLERGEN	Non-Toxin	0.014125147	IL10 non-inducer	Non IL4 inducer
RV2659C	HLA-DRB4*01:01	1	347	358	12	LRYQHAAKG	AALRYQHAAKGR	0.3102	0.06	1.0668	NON-ALLERGEN	Non-Toxin	0.43853513	IL10 non-inducer	Non IL4 inducer
RV3879C	HLA-DQA1*01:01/DQB1*05:01	1	39	56	18	IRNLADARL	GLAPAIRNLADARLGVTL	0.0298	0.47	0.6025	NON-ALLERGEN	Non-Toxin	0.47704596	IL10 non-inducer	Non IL4 inducer
RV3879C	HLA-DQA1*01:01/DQB1*05:01	1	40	56	17	IRNLADARL	LAPAIRNLADARLGVTL	0.0363	0.48	0.5405	NON-ALLERGEN	Non-Toxin	0.15017235	IL10 non-inducer	Non IL4 inducer
RV3872	HLA-DRB3*01:01	1	5	22	18	IAADIGTQV	SHDPIAADIGTQVSDNAL	0.7544	0.21	0.5918	NON-ALLERGEN	Non-Toxin	0.018328218	IL10 non-inducer	Non IL4 inducer
RV3873	HLA-DQA1*01:02/DQB1*06:02	1	81	97	17	AMQATAQAA	QAKTRAMQATAQAAAYT	0.6849	0.43	0.7682	NON-ALLERGEN	Non-Toxin	0.45477448	IL10 non-inducer	Non IL4 inducer

Table S3. Preliminary screening results of linear B-cell epitopes.

Antigen	Sequence	Start position	Score	Allergenicity	Toxicity
RV1736C	HTISTYGPDRVAGFSP	214	0.94	NON-ALLERGEN	Non-Toxin
RV1736C	AVGSWWRYRYDKFGWT	462	0.93	NON-ALLERGEN	Non-Toxin
RV1980C	SIAPNAGLDPVNYQNF	202	0.92	NON-ALLERGEN	Non-Toxin
RV1980C	AATSSTPREAPYELNI	107	0.92	NON-ALLERGEN	Non-Toxin
RV2656C	TPSSTDPTASRAVSWW	53	0.93	NON-ALLERGEN	Non-Toxin
RV2656C	SREIQRRRDAYIRRVV	141	0.81	NON-ALLERGEN	Non-Toxin
RV2659C	GRVYIAPKTFNAKIDA	59	0.97	NON-ALLERGEN	Non-Toxin
RV2659C	ATTAVGTPTMRAHSYS	155	0.9	NON-ALLERGEN	Non-Toxin
RV2879C	LIPLNPTPGSDWDASP	161	0.86	NON-ALLERGEN	Non-Toxin
RV2879C	APAIRNLADARLGVTL	68	0.84	NON-ALLERGEN	Non-Toxin
RV3872	EKMSHDPIAADIGTQV	27	0.87	NON-ALLERGEN	Non-Toxin
RV3872	MTERCLSISHRVRVPE	10	0.84	NON-ALLERGEN	Non-Toxin
RV3873	APLAQEREEDDEDDWD	374	0.93	NON-ALLERGEN	Non-Toxin
RV3873	SSTPVGQLPPAATQTL	220	0.85	NON-ALLERGEN	Non-Toxin

Table S4. Analysis of Physicochemical Properties and MHC Coverage of Antigens with Different Topological Scaffolds.

Topological scaffolds	Antigenicity	Immunogenicity	Toxicity	Allergenicity	Molecular Weight	Theoretical Pi	Instability Index	Aliphatic Index	GRAVY	Solubility	MHCI (world)	MHC II (world)
1	0.6953	7.30139	NO	NO	85084.3	9.5	25.42	74.4	-0.362	0.532	50.24%	99.67%
2	0.6866	6.61139	NO	NO	84837.19	9.57	27.5	75.21	-0.347	0.563	73.57%	99.67%
3	0.6904	6.17249	NO	NO	84874.13	9.47	26.11	74.15	-0.342	0.551	74.45%	99.67%
4	0.6813	6.43769	NO	NO	85145.17	9.56	26.26	71.25	-0.415	0.545	47.90%	99.67%
5	0.6704	6.25079	NO	NO	84995.2	9.52	27.13	75.37	-0.377	0.556	73.56%	99.67%
6	0.6704	6.25079	NO	NO	84995.2	9.52	27.13	75.37	-0.377	0.556	73.56%	99.67%
7	0.6869	6.58709	NO	NO	84692.96	9.48	28.58	75.1	-0.339	0.53	73.57%	99.67%
8	0.6907	6.14819	NO	NO	84729.9	9.36	27.19	74.03	-0.333	0.514	74.45%	99.67%
9	0.6815	6.41339	NO	NO	85000.94	9.46	27.34	71.13	-0.406	0.533	47.27%	99.67%
10	0.6707	6.22649	NO	NO	84850.97	9.42	28.21	75.26	-0.368	0.6707	73.56%	99.67%
11	0.6933	6.39179	NO	NO	83999.08	9.49	25.32	72.53	-0.383	0.53	58.18%	99.67%
12	0.6844	5.70179	NO	NO	83751.97	9.57	27.43	73.36	-0.369	0.557	77.99%	99.67%
13	0.6883	5.26289	NO	NO	83788.91	9.47	26.02	72.28	-0.363	0.542	82.35%	99.67%
14	0.679	5.52809	NO	NO	84059.95	9.55	26.17	69.33	-0.437	0.543	54.72%	99.67%
15	0.668	5.34119	NO	NO	83909.98	9.52	27.06	73.52	-0.398	0.556	75.81%	99.67%
16	0.6906	6.05309	NO	NO	84046.18	9.51	25.93	72.29	-0.392	0.543	57.67%	99.67%
17	0.6847	5.67749	NO	NO	83607.74	9.47	28.52	73.24	-0.36	0.531	77.99%	99.67%
18	0.6886	5.23859	NO	NO	83644.68	9.35	27.11	72.16	-0.354	0.511	82.35%	99.67%
19	0.6793	5.50379	NO	NO	83915.72	9.45	27.27	69.22	-0.428	0.531	54.18%	99.67%
20	0.6683	5.31689	NO	NO	83765.75	9.42	28.15	73.41	-0.389	0.538	75.81%	99.67%

Table S5. Vaccines and Their Physicochemical Properties During Agonist Screening.

Vaccine	TLR2	TLR4	AA length	Antigenicity	Immunogenicity	Toxicity	Allergenicity	Molecular Weight	Theoretical pI	Instability Index	Aliphatic Index	GRAVY	Solubility
**1**		RPIL	790	0.6883	5.26289	Non-Toxin	NON-ALLERGEN	83788.91	9.47	26.02	72.28	-0.363	0.542
**2**		RPFE(RV2450C)	832	0.7632	8.13442	Non-Toxin	NON-ALLERGEN	87786.64	9.52	31.29	67.25	-0.426	0.455
**3**		HBHA	847	0.8382	7.09474	Non-Toxin	NON-ALLERGEN	88778.77	9.56	28.37	65.73	-0.574	0.494
**4**		CTB	796	0.8856	4.37879	Non-Toxin	NON-ALLERGEN	82942.65	9.84	26.29	61.07	-0.576	0.577
**5**		RS-09	667	0.7444	4.2288	Non-Toxin	NON-ALLERGEN	71040.22	9.81	27.89	66.27	-0.465	0.488
**6**	ESAT-6		755	0.7212	4.43589	Non-Toxin	NON-ALLERGEN	80252.3	9.65	28.37	66.85	-0.443	0.488
**7**	PSMa4		694	0.7687	5.01179	Non-Toxin	NON-ALLERGEN	72940.62	9.89	24.12	66.17	-0.466	0.577

**8**	PorB		761	0.7249	4.65171	Non-ToxinNON-ALLERGEN	80551.99	9.76	25.53	69.5	-0.395	0.536
**9**	Pam2Cys		681	0.7499	4.42925	Non-ToxinNON-ALLERGEN	72827.27	9.82	27.55	66.61	-0.452	0.481
**10**	ESAT-6	RPIL	885	0.6716	5.09259	Non-ToxinNON-ALLERGEN	93674.77	9.29	27.51	72.27	-0.352	0.496
**11**	ESAT-6	RPFE(RV2450C)	927	0.7403	8.01039	Non-ToxinNON-ALLERGEN	97672.5	9.34	31.81	67.76	-0.409	0.431
**12**	ESAT-6	HBHA	914	0.6925	7.06209	Non-ToxinNON-ALLERGEN	97823.85	9.26	31.9	71.48	-0.456	0.439
**13**	ESAT-6	CTB	859	0.6904	4.19199	Non-ToxinNON-ALLERGEN	91972.76	9.55	30.31	68.65	-0.434	0.453
**14**	ESAT-6	RS-09	762	0.7171	4.35009	Non-ToxinNON-ALLERGEN	80926.07	9.65	28.71	67.01	-0.439	0.483
**15**	PSMa4	RPIL	824	0.7078	5.66849	Non-ToxinNON-ALLERGEN	86363.08	9.54	23.86	72.1	-0.364	0.589
**16**	PSMa4	RPFE(RV2450C)	866	0.7797	8.58629	Non-ToxinNON-ALLERGEN	90360.81	9.61	28.65	67.27	-0.424	0.513
**17**	PSMa4	HBHA	853	0.729	7.63799	Non-ToxinNON-ALLERGEN	90512.16	9.54	28.69	71.25	-0.475	0.514
**18**	PSMa4	RS-09	702	0.7641	5.02349	Non-ToxinNON-ALLERGEN	73743.5	9.87	24.67	66.25	-0.465	0.567
**19**	PorB	RPIL	891	0.675	5.30841	Non-ToxinNON-ALLERGEN	93974.46	9.45	25.09	74.5	-0.311	0.527
**20**	PorB	RPFE(RV2450C)	933	0.7432	8.22621	Non-ToxinNON-ALLERGEN	97972.19	9.5	29.47	69.91	-0.37	0.464
**21**	PorB	HBHA	920	0.6957	7.27791	Non-ToxinNON-ALLERGEN	98123.54	9.43	29.53	73.64	-0.416	0.478
**22**	PorB	RS-09	769	0.7212	4.66341	Non-ToxinNON-ALLERGEN	81354.88	9.74	26.02	69.54	-0.396	0.524
**23**	Pam2Cys	RPIL	811	0.6911	5.08595	Non-ToxinNON-ALLERGEN	86249.73	9.49	26.74	72.56	-0.35	0.531
**24**	Pam2Cys	RPFE(RV2450C)	853	0.7649	8.00375	Non-ToxinNON-ALLERGEN	90247.46	9.54	31.46	67.64	-0.413	0.445
**25**	Pam2Cys	HBHA	840	0.7132	7.05545	Non-ToxinNON-ALLERGEN	90398.81	9.47	31.55	71.69	-0.464	0.445
**26**	Pam2Cys	RS-09	688	0.7451	4.34345	Non-ToxinNON-ALLERGEN	73501.04	9.82	27.93	66.79	-0.447	0.475

Table S6. Structural Scores of the RP14914P Vaccine Model After Optimization.

Model	GDT-HA	RMSD	MolProbity	Clash score	Poorrotamers	Rama favored	Z value（Before optimization）	Z value（ After optimization）	ERRAT(Overall Quality Factor)
MODEL 5	0.8606	0.67	0.91	1.6	0.2	98.8	-5.97	-6.21	80.4813
MODEL 2	0.8727	0.638	0.812	1.1	0.2	98.6			
MODEL 4	0.8701	0.667	0.812	1.1	0	98.4			
MODEL 3	0.8684	0.65	0.871	1.4	0.2	98.1			
MODEL 1	0.8707	0.651	0.883	1.5	0	98			
Initial	1	0	2.105	2.3	3.8	85.3			

Table S7. Discontinuous B-cell epitopes in the RP14914P structure predicted by ElliPro.

NO	Residues	Number of Residues	Score
1	A:D659, A:D660, A:E661, A:D662, A:D663, A:W664, A:D665, A:K666	8	0.962
2	A:R495, A:E496, A:A497, A:P498, A:Y499	5	0.908
3	A:E292, A:A293, A:P294, A:Y295	4	0.906
4	A:A560, A:T561, A:T562, A:A563, A:V564, A:G565, A:T566, A:P567 A:T568 A:M569	10	0.887

5	A:E754, A:D755, A:A756, A:G757, A:F758, A:D759, A:P760, A:N761, A:L762, A:P763, A:P764, A:P765, A:L766, A:A767, AP768, AD769, A:F770, A:L771, A:S772, A:P773, A:P774, A:A775 A:E776 A:E777	24	0.885
6	A:F275, A:A276, A:V277, A:T278, A:N279, A:D280, A:G281, A:G282, A:P283, A:G284, A:P285, A:G286, A:S287, A:S288, A:T289 A:P290 A:R291	17	0.859
7	A:V706, A:T707, A:T708, A:S709, A:P710, A:A711, A:G712, A:I713, A:A714, A:N715, A:A716, A:D717, A:D718, A:A719, A:G720, A:L721, A:D722, A:P723, A:N724, A:A725, A:A726, A:A727, A:G728, A:P729, A:D730, A:A731, A:V732, A:G733, A:F734, A:D735, A:P736, A:N737, A:L738, A:P739, A:P740, A:A741, A:P742, A:D743, A:A744, A:A745, A:P746, A:V747, A:D748 A:T749 A:P750 A:P751 A:A752 A:P753	48	0.857
8	A:E296, A:L297, A:N298, A:I299, A:T300, A:S301, A:A302, A:T303, A:Y304, A:G305, A:P306, A:G307, A:P308, A:G309, A:A310, A:P311, A:T312, A:L313, A:A314, A:A315, A:A316, A:V317, A:E318, A:W319, A:P320, A:M321, A:A322, A:G323, A:T324, A:G325, A:P326, A:G327, A:P328, A:G329, A:A330, A:A331 A:L332	37	0.854
9	A:E500, A:L501, A:N502, A:I503, A:K504	5	0.828
10	A:R570, A:A571, A:H572, A:S573	4	0.826
11	A:T160, A:R161, AA162, A:H163, A:R165, A:K166	6	0 799
12	A:A488, A:A489, A:T490, A:S491, A:S492, A:T493, A:P494	7	0 771
13	A:E647, A:K649, A:A650, A:P651, A:L652, A:A653, A:Q654, A:E655	8	0 763
14	A:K667, A:S668, A:S669, A:T670, A:P671, A:V672, A:G673, A:Q674, A:L675, A:P676, A:P677, A:A678, A:A679, A:T680, A:Q681 A:T682 A:L683 A:E684	18	0.750
